# *C-Jun* drives melanoma progression in *PTEN* wild type melanoma cells

**DOI:** 10.1038/s41419-019-1821-9

**Published:** 2019-08-05

**Authors:** Melanie Kappelmann-Fenzl, Claudia Gebhard, Alexander O. Matthies, Silke Kuphal, Michael Rehli, Anja Katrin Bosserhoff

**Affiliations:** 1 Institute of Biochemistry (Emil-Fischer Center), Friedrich-Alexander University Erlangen-Nürnberg, Erlangen, Germany; 2 Faculty of Applied Health Care Sciences, University of Applied Science, Deggendorf, Germany; 3 Department of Internal Medicine III, University Hospital Regensburg, Regensburg, Germany; 4 Regensburg Center for Interventional Immunology (RCI), University Regensburg and University Medical Center Regensburg, 93053 Regensburg, Germany; 5 Comprehensive Cancer Center (CCC)-EMN, Erlangen, Germany

**Keywords:** Oncogenes, Melanoma, Cell signalling

## Abstract

Due to the critical impact of active AP-1 transcription factors in melanoma, it is important to define their target genes and to identify and ultimately inhibit oncogenic signals. Here we mapped the genome-wide occupancy of the AP-1 family member *c-Jun* in different melanoma cells and correlated AP-1 binding with transcriptome data to detect genes in melanoma regulated by *c-Jun*. Our analysis shows that *c-Jun* supports the malignant phenotype by deregulating genes in cancer-relevant signaling pathways, such as mitogen-activated protein kinase (MAPK) and phosphatidylinositol-3-kinase (PI3K) pathways. Moreover, we demonstrate that the importance of *c-Jun* depends on melanoma stage and mutation status of the tumor suppressor *PTEN*. Our study reveals that activation of *c-Jun* overrules the tumor suppressive effect of *PTEN* in early melanoma development. These findings help to understand the relevance of *c-Jun* within cancer pathways in different melanoma cell types, especially in relation to MAPK and PI3K pathways, which are commonly deregulated in melanomas. Consequently, targeting *c-Jun* in *PTEN*^+^ melanoma cells may represent a promising therapeutic strategy to inhibit survival of melanoma cells to prevent the development of a metastatic phenotype.

## Introduction

Melanoma is a highly aggressive and heterogeneous type of cancer, and its incidence is growing faster than any other cancer entity. In recent decades, many altered pathways regulating the development and progression of melanoma and the high migratory and invasive potential of melanoma cells have been identified, but a detailed molecular understanding of this disease is largely lacking.

One crucial step in melanoma development and progression is the deregulation of cancer-supporting transcription factors, especially activating protein-1 (AP-1) transcription factors, including the *c-Jun*, *JDP*, *c-Fos*, *FRA*, and *MAF* subfamilies^1^. AP-1 proteins bind to the classical palindromic recognition sequence 5′-TGA(C/G)TCA-3′ and regulate target gene expression, leading to deregulation of cancer-relevant signaling pathways. Thus, AP-1 transcription factor dimers play an important role in different cancer types, including malignant melanoma^1–5^. A main characteristic of AP-1 complexes in the cell is their heterogeneity in dimer composition. This heterogeneity is caused by the fact that multiple AP-1 subunits can be expressed simultaneously, and different dimer compositions lead to the transcriptional regulation of different target genes. Various studies demonstrated that differences in AP-1 dimer compositions cause altered specificity in their binding site selection^6^. However, it remains unclear which direct target genes of AP-1 homodimer or heterodimer cause the functional effects that support melanomagenesis.

The AP-1 family member *c-Jun* is a main regulator of melanoma progression^4,7,8^, acts by regulating target genes supporting proliferation and migration of cancer cells, and thus promotes the malignant phenotype. We have previously demonstrated that the microRNA miR-125b directly regulates the transcription factor *c-Jun*, affecting the proliferative and migratory potential of melanoma cells^9^. Furthermore, we identified an alternative regulatory pathway of *c-Jun* in melanoma cells leading to upregulation of *c-Jun* activity via the loss of the cell-adhesion molecule E-cadherin^10,11^. Despite its role in the aforementioned pathophysiological processes, only a few specific *c-Jun* target genes have been identified to date. Previous chromatin immunoprecipitation (ChIP) studies focusing on AP-1/*c-Jun* in nonmelanoma cell types identified a few cancer-related target genes of *c-Jun*. Deregulation of *c-Jun* is one of the most important events in malignant melanoma and many other cancer entities, but the functional relevance of *c-Jun* deregulation and its molecular effects on target gene expression have not been determined in detail to date.

Another crucial event supporting cancer cell survival is the upregulation of PI3K/AKT signaling activity in various cancer types, which can largely be attributed to deregulation of the *AKT* negative regulator, phosphatase, and tensin homolog (*PTEN*) phosphatase^12^. Mutations in the *PTEN* gene and thus the loss of this tumor suppressor protein are prevalent in melanoma and lead to upregulation of *AKT* activity. Activated *AKT* protects cells from apoptosis by phosphorylating and inactivating proapoptotic substrates^13^. Mutational inactivation of *PTEN* frequently occurs in human cancers; however, early molecular tumor-promoting mechanisms in *PTEN* expressing (*PTEN*^+^) cancer cells, such as those in early developing melanoma, remain unclear.

In this study, we demonstrate that *c-Jun* upregulation in melanoma cells depends on melanoma stages (primary tumor (PT) vs. metastasis (MET)) and *PTEN* expression status.

## Results

### *C-Jun* and the tumor suppressor *PTEN* exhibit a positive correlation in melanoma cells

Given that melanomas gain different properties during their progression, we selected six different melanoma cell lines (Sbcl-2, WM3211, WM793, WM1366, WM1158, WM9) categorized by tumor stage (PT, MET) and their previously described BRAF and *PTEN* mutation status^14^. All cell line characteristics are shown in Supplementary Table 1, and the described mutations of each cell line were confirmed by our RNA sequencing data (Supplementary Table 1, Supplementary Fig. 2). We first performed functional assays to analyze the proliferative (Supplementary Fig. 1Ai) and/or migratory potential (Supplementary Fig. 1Aii) of four melanoma cell lines exhibiting different tumor stages, BRAF mutation statuses, and *PTEN* deletions. Interestingly, based on the mentioned characteristics, a distinct classification based on proliferative and/or migratory behavior was not possible. Thus, we further focused on *c-Jun*-dependent differences of gene expression among the analyzed melanoma cell lines.

**Fig. 1:**
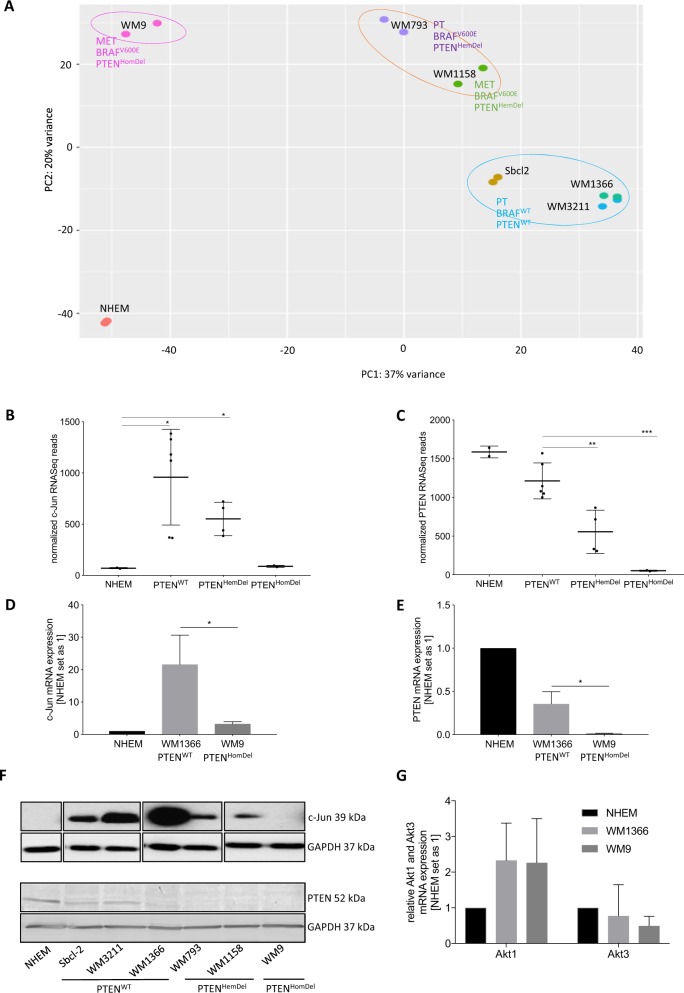
*C-Jun* and *PTEN* exhibit a positive correlation in malignant melanoma. **a** Low-dimensional representations of the expression profiles of the RNA-Seq data (NHEM, Sbcl2, WM3211, WM1366, WM793, WM1158, WM9) by principal component analyses (PCA) (DESeq2 in R, Bioconductor). RNA-Seq data visualization revealed sample clustering based on *PTEN* and BRAF expression status. **b** Normalized *c-Jun* RNA-Seq reads. **c** Normalized *PTEN* RNA-Seq reads. **d** *c-Jun* mRNA expression analysis via qRT-PCR normalized to ß-actin. **e** *PTEN* mRNA expression analysis via qRT-PCR normalized to ß-actin. **f** Western blot analysis revealing *c-Jun* and *PTEN* protein levels in different melanoma cell lines compared with NHEMs. C-Jun Western blot positions are adapted and the original Western Blot is shown in Supplementary Fig. 1B. GAPDH serves as loading control. **g** *AKT1* and *AKT3* mRNA expression analysis via qRT-PCR normalized to ß-actin. The box-plots show the mean ± s.d. of read counts of two independent experiments (***P < 0.001; **P < 0.01; *P < 0.5). The bars show the mean ± s.d. of three independent experiments; measurements were performed in triplicate (*P < 0.5)

We first analyzed differentially expressed genes in malignant melanoma by performing RNA-Sequencing (RNA-seq) with all aforementioned melanoma cell lines and normal human epidermal melanocytes (NHEM). We assigned tumor stage and mutation status to our samples and performed *principal component analyses* (PCA) to construct low-dimensional representations of the expression profiles of the complete RNA-Seq data set. The PCA results clearly demonstrated sample clustering based on *PTEN* and BRAF status (Fig. 1a).

Analysis of mRNA expression levels of both *c-Jun* and *PTEN* in all used melanoma cell lines (compared to NHEMs) revealed high *c-Jun* expression levels in *PTEN*^WT^ cells that were gradually reduced in *PTEN*^HemDel^ and *PTEN*^HomDel^ cells (Fig. 1b). *PTEN* mRNA expression follows the corresponding copy numbers of the used cell lines, demonstrating high *PTEN* mRNA levels in NHEMs and *PTEN*^WT^ that continuously decrease in *PTEN*^HemDel^ and *PTEN*^HomDel^ (Fig. 1c). We exemplarily confirmed *c-Jun* and *PTEN* mRNA expression via qRT-PCR analysis in one *PTEN*^WT^ (WM1366) and one *PTEN*^HomDel^ (WM9) melanoma cell line (Fig. 1d, e) compared to NHEMs. In accordance with these findings, Western blot analyses of *PTEN*^WT^ melanoma cells exhibited high *c-Jun* protein levels, which decreased with the gradual loss of *PTEN* (Fig. 1f, Supplementary Fig. 1B). Due to the fact that *PTEN* is known to inhibit *AKT* activity (predominantly *AKT3* in melanoma) and thus promotes melanoma cell apoptosis, loss of *PTEN* is a crucial event during melanoma development and progression. Hence, we further analyzed *AKT1* and *AKT3* mRNA expression by qRT-PCR in both *PTEN*^+^ and *PTEN*^−^ melanoma cells. Interestingly, no differences in *AKT* expression between *PTEN*^WT^ and *PTEN*^HomDel^ cells were detected (Fig. 1g), supporting the role of loss of *PTEN* on *AKT* activity.

Consequently, we hypothesize that *PTEN*^+^ melanoma cells in early melanoma development utilize *c-Jun* to overcome the tumor-suppressive effect of *PTEN*.

### *C-Jun* significantly influences gene expression in malignant melanoma

**Fig. 2:**
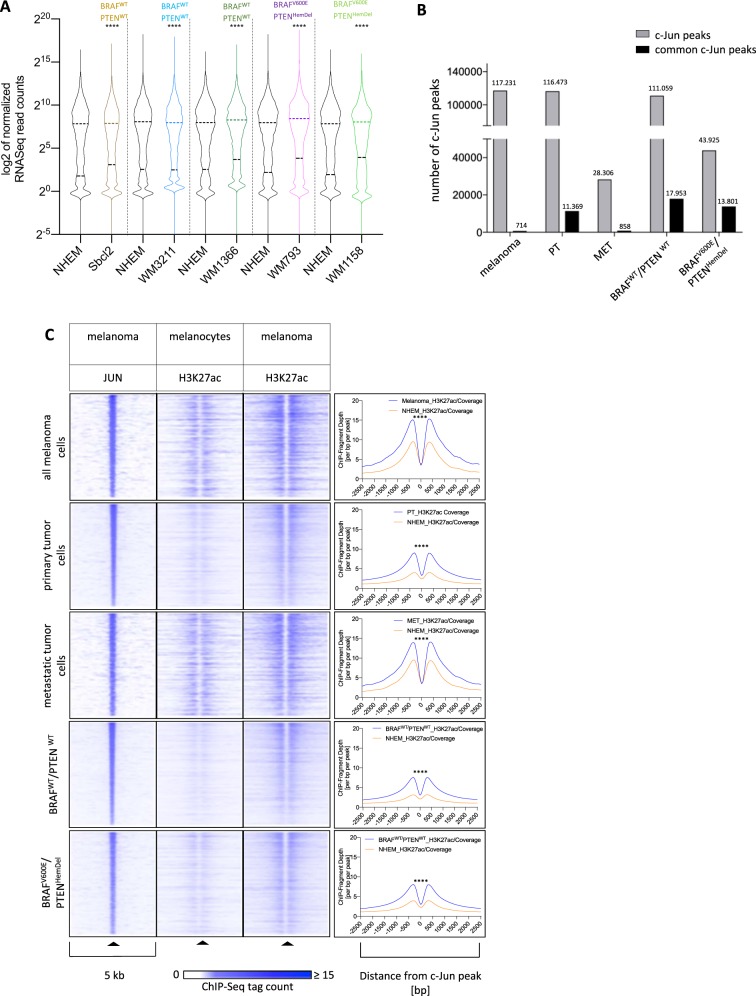
*C-Jun* significantly influences gene expression alteration in malignant melanoma. **a** All sequenced melanoma cell lines show significant differences in gene expression due to the regulatory activity of *c-Jun* compared to NHEMs. Violin blots display the interquartile ranges (25–75%) with an intersection as the median; Significantly different distributions in pairwise comparisons are indicated (****P < 0.0001, Wilcoxon test, two-sided). **b** Clustering of *c-Jun* peak sets by *PTEN* expression status revealed a correlation between *c-Jun* activity and *PTEN* expression. **c** Heat maps and histograms of ChIP-Seq tag counts of the histone acetylation status (H3K27ac) in a 5-kb wide range around *c-Jun* peaks. Regions centered on *c-Jun*-bound regions were clustered according to their H3K27ac ChIP-Seq profiles in melanoma and NHEMs, illustrating a high acetylation rate around the genomic *c-Jun* peaks in melanoma and a low rate in NHEMs (****P < 0.0001, Wilcoxon test, paired, two-sided)

To further explore the *c-Jun* influences gene expression in malignant melanoma, we mapped its binding sites using ChIP-Sequencing (ChIP-seq). First, we identified genes associated with *c-Jun* peaks in each melanoma cell line and compared their mRNA expression to normal NHEMs. Our analysis showed a significant induction of *c-Jun* peak-associated genes in each sequenced *c-Jun* expressing melanoma cell line (Sbcl2, WM3211, WM1366, WM793, and WM1158), suggesting a positive regulatory activity of *c-Jun* (Fig. 2a). Moreover, analysis of the resulting *c-Jun* ChIP-Seq peaks confirmed the melanoma cell line clustering detected via RNA-Seq (Fig. 1a). Most interestingly, our *c-Jun* ChIP-Seq data clearly revealed differences in DNA-binding activity of *c-Jun* based on the cell line-specific *PTEN* copy number alteration. Grouping of *c-Jun* peak sets by *PTEN* expression status resulted in 17.953 common *c-Jun* peaks in *PTEN*^WT^ (Sbcl-2, WM3211, WM1366) and 13.801 in *PTEN*^HemDel^ (WM793, WM1158) cell lines (Fig. 2b). The abundance and overlap of *c-Jun* peaks across melanoma cells confirm the relationship between *c-Jun* activity and *PTEN* expression status as observed on transcript level.

To further study the impact of *c-Jun* on the activity status of bound regulatory elements, we compared the local deposition of H3K27ac, a histone modification associated with active regulatory sites, at *c-Jun* peaks (Supplementary Table 2) in melanoma cells compared to NHEMs. As shown in heatmap representations and corresponding histograms, *c-Jun*-bound regions show a higher local acetylation in melanoma compared to melanocytes (Fig. 2c).

Taken together, our data demonstrate that gene expression differences between all analyzed melanoma cell lines depend on *c-Jun* and *PTEN* expression/activity. These findings indicate that *c-Jun* plays a crucial role in early melanoma development.

### Functional annotation reveals an enrichment of PI3K/AKT signaling in *PTEN*^+^/*c-Jun*^+^ melanoma cells

**Fig. 3:**
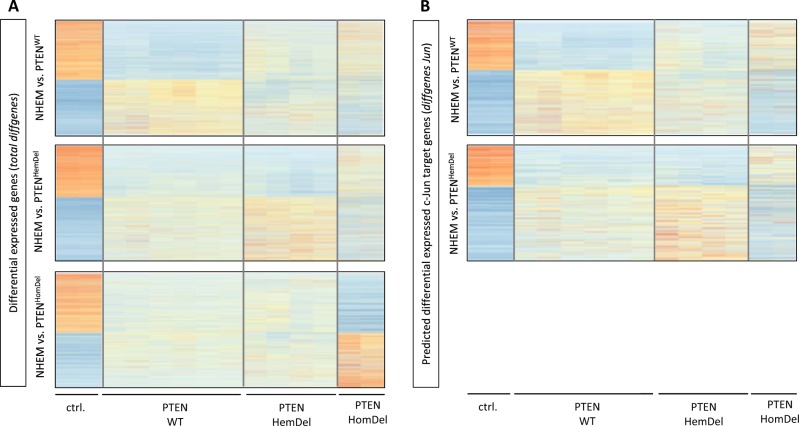
Gene set enrichment analysis resulted in an enrichment of PI3K/AKT signaling in *PTEN*-expressing and *c-Jun*-expressing melanoma cells. **a** Differential expression analysis via DESeq2 (R, Bioconductor). Heat maps illustrate RNA-Seq differential expression in *PTEN*^WT^, *PTEN*^HemDel^, or *PTEN*^HomDel^ cells compared to NHEMs. **b** RNA-Seq differential expression results combined with *c-Jun* ChIP-Seq peaks illustrated as heat maps of differentially expressed genes regulated by *c-Jun* in *PTEN*^WT^ and *PTEN*^HemDel^ cells

As a next step, we were interested in the functional relevance of all differentially expressed genes (based on RNA-Seq) and the subset of differentially expressed genes associated with a *c-Jun* peak. We performed differential expression analysis of the RNA-Seq data described above and compared the gene expression status of *PTEN*^WT^, *PTEN*^HemDel^, or *PTEN*^HomDel^ cells with NHEMs. The comparison resulted in three different gene sets with differentially expressed genes represented as heat maps (Fig. 3a, *total diffgenes*). These gene sets were subsequently merged with *c-Jun* ChIP-Seq peak annotation data to identify those genes differentially expressed, which are regulated by *c-Jun* (Fig. 3b, *diffgenes Jun*). The amount of total differentially expressed genes and differentially expressed genes associated with a *c-Jun* peak of all generated gene sets are illustrated in Supplementary Table 3. Regarding *PTEN*^HomDel^ cells (lacking *c-Jun*), we further explored the total differentially expressed genes.

These newly generated data sets, including the total differentially expressed genes and differentially expressed genes associated with a *c-Jun* peak, were used to perform detailed functional annotation clustering with the bioinformatic tools *Database for Annotation*, *Visualization and Integrated Discovery* (DAVID; v 6.8) *and Gene Set Enrichment Analysis* (GSEA) tool, respectively, to identify enriched gene sets within each data set and their molecular functions.

Functional annotation analysis via DAVID^15,16^ resulted in several melanoma-/cancer-specific enriched *Kyoto Encyclopedia of Genes and Genomes* (KEGG) pathways (Table 1). Interestingly, functional annotation of the differentially expressed genes associated with a *c-Jun* peak yielded similar enriched pathways in *PTEN*^WT^ and *PTEN*^HemDel^ cells as the total differentially expressed genes of *PTEN*^HomDel^ cells. Our analysis revealed *c-Jun* to be a regulator of genes involved in the PI3K/AKT signaling pathway in *PTEN*^+^ melanoma cells, indicating that *c-Jun* mainly supports anti-apoptotic and pro-survival processes in early (*PTEN*^WT^ and *PTEN*^HemDel^) melanoma development.

**Table 1:** DAVID functional annotation analysis. Functional annotation analysis of RNA-Seq data via DAVID of the five generated gene sets (NHEM vs. *PTEN*^WT^, *PTEN*^HemDel^, *PTEN*^HomDel^, *PTEN*^WT^*c-Jun* or *PTEN*^HemDel^*c-Jun*) depicting similar results in terms of enriched KEGG pathways

*total diffgenes*		
KEGG pathway	Size	*P*-value
NHEM-*PTEN*^WT^		
Pathways in cancer	114	3.23E-05
PI3K-Akt signaling pathway	90	7.94E-03
Rap1 signaling pathway	71	6.23E-06
Focal adhesion	68	2.43E-05
Endocytosis	68	1.69E-02
Proteoglycans in cancer	67	1.66E-05
Ras signaling pathway	62	9.68E-03
Lysosome	61	5.40E-13
NHEM-*PTEN*^HemDel^		
Pathways in cancer	88	1.97E-06
PI3K-Akt signaling pathway	66	4.05E-03
HTLV-I infection	57	1.95E-04
Rap1 signaling pathway	55	2.00E-06
Lysosome	51	4.36E-14
Focal adhesion	49	1.14E-04
Neuroactive ligand–receptor interaction	49	4.92E-02
Cytokine–cytokine receptor interaction	48	2.79E-03
NHEM-*PTEN*^HomDel^		
Pathways in cancer	54	4.46E-02
PI3K-Akt signaling pathway	48	4.88E-02
Rap1 signaling pathway	39	7.93E-04
Focal adhesion	34	1.18E-02
Axon guidance	31	2.12E-05
Lysosome	26	9.01E-04
Cell cycle	24	5.99E-03
Homologous recombination	8	3.03E-02
Other types of O-glycan biosynthesis	8	4.24E-02

*diffgenes Jun*		
NHEM-*PTEN*^WT^_Jun		
Pathways in cancer	45	3.59E-05
PI3K-Akt signaling pathway	35	2.76E-03
Rap1 signaling pathway	32	2.39E-06
Focal adhesion	31	4.63E-06
Proteoglycans in cancer	25	8.45E-04
MAPK signaling pathway	25	1.86E-02
Endocytosis	25	2.14E-02
Ras signaling pathway	24	8.73E-03
NHEM-*PTEN*^HemDel^_Jun		
Pathways in cancer	31	5.11E-04
PI3K-Akt signaling pathway	24	1.19E-02
Rap1 signaling pathway	20	7.85E-04
Cytokine–cytokine receptor interaction	20	2.30E-03
Focal adhesion	19	1.60E-03
Ras signaling pathway	18	9.70E-03
cAMP signaling pathway	17	6.17E-03
Hippo signaling pathway	15	3.02E-03

The GSEA tool utilizes the expression status (read counts of RNA-Seq) for functional annotation clustering and thus identifies genes that are upregulated or downregulated. GSEA results of our RNA-Seq data are depicted as enrichment plots and the corresponding *Blue-Pink O’ Gram in the Space of the Analyzed GeneSets* of *Pathways in cancer* of all five comparisons (NHEM vs. *PTEN*^WT^, *PTEN*^*WT*^*c-Jun*, *PTEN*^HemDel^, *PTEN*^*HemDel*^*c-Jun*, or *PTEN*^HomDel^, Supplementary Fig. 3). The outcome is represented in *Profile of the Running ES Score & Positions of GeneSet Members on the Rank Ordered List*. Similar to our previous functional annotation results, we observed *c-Jun*-dependent regulation of PI3K/AKT signaling members in *PTEN*^+^ melanoma cells (Supplementary Fig. 3; red arrows).

As a next step, we assigned biological meaning to the *c-Jun* ChIP-Seq peaks in noncoding genomic regions analyzing the peak annotations by the *Genomic Regions Enrichment of Annotations Tool* (GREAT), a bioinformatic tool that predicts functions of cis-regulatory regions. We used *c-Jun* peaks of genomic regions detected by *c-Jun* ChIP-Seq in *PTEN*^WT^ and *PTEN*^HemDel^ cells. GREAT analysis resulted in an enrichment of several different cancer-relevant pathways, such as PI3K/AKT in both melanoma subgroups *PTEN*^WT^ and *PTEN*^HemDel^, confirming our prior findings.

Furthermore, the identified gene associations of the *c-Jun* ChIP-Seq peaks of *PTEN*^WT^ and *PTEN*^HemDel^ cells were adjusted to the corresponding RNA-Seq differential expression results. These analyses revealed a deregulation of 1084 *c-Jun*-associated genes in *PTEN*^WT^ cells (among overall 4188 differentially expressed genes in *PTEN*^WT^ compared to NHEMs) and 736 (among overall 2764 differentially expressed genes in *PTEN*^HemDel^ compared to NHEMs) in *PTEN*^HemDel^ cells (Supplementary Table 3, *diffgenes c-Jun*). Functional network analyses via *STRING 10.5* (Search Tool for the Retrieval of Interacting Genes/Proteins) of both gene sets, including genes regulated by *c-Jun* in *PTEN*^WT^ and *PTEN*^HemDel^, respectively, also revealed an enrichment of the KEGG pathways similar to those identified by DAVID, GSEA, and GREAT. The number of gene set members and false discovery rates are presented in Supplementary Fig. 4.

In summary, numerous differentially expressed genes in *PTEN*^WT^ and *PTEN*^HemDel^ cells belonging to the PI3K/AKT-signaling pathway are regulated by *c-Jun* (Supplementary Fig. 5, STRING; red: members of PI3K-signaling system), indicating again that *c-Jun* plays a crucial role in early melanoma development to promote survival in the presence of *PTEN*.

### *C-Jun* directly regulates PI3K/AKT members in *PTEN*-positive melanoma cells

**Fig. 4:**
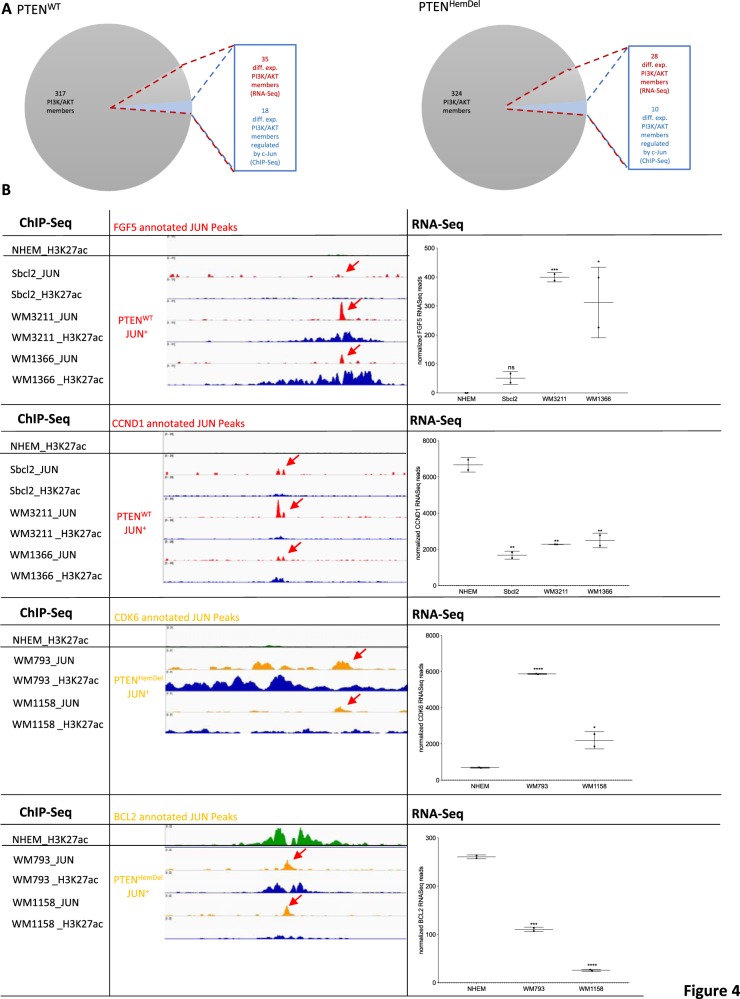
C-Jun directly regulates PI3K/AKT members in *PTEN*-positive melanoma cells. **a** Differentially expressed *PI3K/AKT* members (*PTEN*^WT^: 35/352; *PTEN*^HemDel^: 28/352; RNA-Seq) overlap with *c-Jun-*regulated ones (*PTEN*^WT^: 18/35; *PTEN*^HemDel^: 10/28; ChIP-Seq). **b** Expression status (RNASeq) of four differentially expressed PI3K/AKT-related genes (*PDGFB* and *CCND1* regulated by *c-Jun* in *PTEN*^WT^, *CDK6* and *BCL2* regulated by *c-Jun* in *PTEN*^HemDel^), the associated *c-Jun* peaks (red arrows; ChIP-Seq) and the H3K27 acetylation status around the *c-Jun* peaks (blue), illustrating the differences in transcriptionally active genomic regions between NHEMs and melanoma cell lines. The box-plots show the mean ± s.d. of read counts of two independent experiments (****P < 0.0001; ***P < 0.001; **P < 0.01; *P < 0.5)

Next, we confirmed the identified *c-Jun*-regulated PI3K/AKT-related targets by visualizing *c-Jun* and H3K27 ChIP-Seq peaks and the corresponding RNA-Seq expression data in the IGV Browser^17^. We identified several differentially expressed PI3K/AKT-related genes in *PTEN*^HomDel^ cells, which are regulated by *c-Jun* in *PTEN*^+^ cells. Figure 4a shows the overlap of the identified differentially expressed PI3K/AKT-related genes (RNA-Seq) and the *c-Jun*-regulated genes detected by ChIP-Seq in both subgroups, *PTEN*^WT^ (9.94% diff. exp.; 5.11% *c-Jun* regulated) and PTEN^HemDel^ (7.95% differentially expressed; 2.84% *c-Jun* regulated). Figure 4b displays the expression status of four differentially expressed PI3K/AKT-related genes (*PDGFB* and *CCND1* regulated by *c-Jun* in *PTEN*^WT^, *CDK6*, and *BCL2* regulated by *c-Jun* in *PTEN*^HemDel^), the associated *c-Jun* peaks (red arrows; ChIP-Seq) and the H3K27 acetylation status around the *c-Jun* peaks (blue), illustrating the differences in transcriptionally active genomic regions between NHEMs and melanoma cell lines. Moreover, we confirmed our findings by quantitative mRNA expression analysis of the identified differentially expressed *c-Jun* target genes (FGF5, CCND1, EGFR, PDGFB, LAMB3) after *c-Jun* siRNA transfection (Supplementary Fig. 3B). Our data clearly reveal the potential of the transcription factor *c-Jun* to either upregulated or downregulate target gene expression. All identified differentially expressed genes and those regulated by *c-Jun* of the analyzed melanoma subsets are presented in Supplementary Table 4. The previously described functions of each identified *c-Jun* regulated differentially expressed gene in melanoma are depicted in Supplementary Table 5.

### Validation of *c-Jun*-dependent anti-apoptotic and pro-survival effects in early melanoma cells

**Fig. 5:**
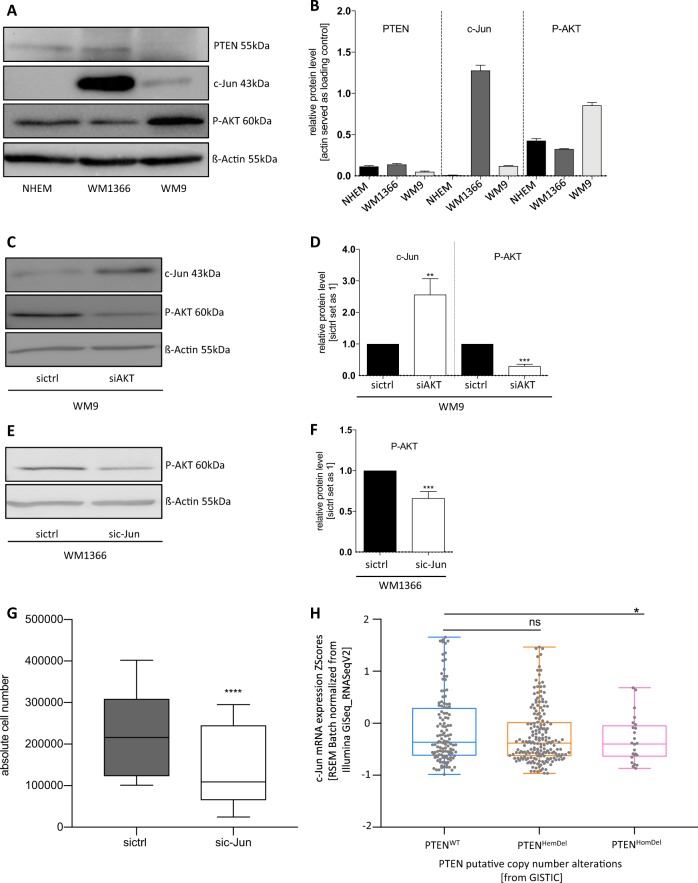
*c-Jun-*dependent anti-apoptotic and pro-survival behavior of melanoma cells. **a** Western blot analysis revealing *PTEN*, *c-Jun*, and P-*AKT* protein levels in WM1366 and WM9 melanoma cell lines compared with NHEMs. ß-actin served as loading control. **b** Densitometry of the *PTEN*, *c-Jun*, and P-*AKT* protein level compared to ß-actin. **c** Western blot analysis after *AKT*-siPool transfection of WM9 melanoma cells revealed increased *c-Jun* protein expression compared to sictrl-transfected cells. ß-actin served as loading control. **d** Densitometry of the *c-Jun* and P-*AKT* protein level after *AKT* knockdown compared to ß-actin. **e** Western blot analysis after *c-Jun*-siPool transfection show an decreased *AKT* activity (P-*AKT*). ß-actin served as loading control. **f** Densitometry of the P-*AKT* protein level after *c-Jun* knockdown compared to ß-actin. **g** Total cell amount after *c-Jun* knockdown by siRNA transfection compared to the amount of sictrl-transfected cells. A significant decrease of cell survival in the si*c-Jun* knockdown samples is depicted. **h** Analysis of TGCA sequencing data of a cohort of 471 melanoma patients. Data were categorized by the *PTEN* copy number alteration (*PTEN*^WT^, *PTEN*^HemDel^, *PTEN*^HomDel^) and further analyzed regarding to the *c-Jun* expression status by cBioPortal (*P < 0.05, Welch's test, one-sided). The box-plots show the mean ± s.d. of three independent experiments; measurements were performed in triplicate (****P < 0.0001; ***P < 0.001; **P < 0.01)

To validate our findings, we performed knockdown experiments. First, we determined the total protein amount of *PTEN*, *c-Jun*, and phospho-*AKT* in *PTEN*^+^/*c-Jun*^+^ (WM1366) or *PTEN*^−^/*c-Jun*^−^ (WM9) melanoma cells by Western blot analysis (Fig. 5a, b). We observed increased phospho-*AKT* (P-*AKT*) in WM9 melanoma cells lacking *PTEN* and *c-Jun* expression. Thus, we hypothesized that *c-Jun* expression and transcriptional activity promotes survival in *PTEN*-expressing melanoma cells. Interestingly, *AKT*-siPool transfection in *PTEN*−/*c-Jun*^-^ WM9 melanoma cells to mimic *PTEN* re-expression increased *c-Jun* protein expression (Fig. 5c, d). Moreover, we could validate our prior findings after *c-Jun*-siPool transfection in WM1366. Western blot analysis depicted a decreased *AKT* activity (P-*AKT*) (Fig. 5e, f) and inhibition of *c-Jun* after siRNA transfection resulted in a significant decrease in cell number compared to sictrl-transfected cells (Fig. 5g). Transfection efficiencies are presented in Supplementary Fig. 6.

Importantly, these findings could also be validated by analyzing TCGA-sequencing data (https://www.cancer.gov/tcga) of a cohort of 471 melanoma patients (TCGA, Project ID: TCGA-SKCM). These data were categorized by the *PTEN* copy number alteration (*PTEN*^WT^, *PTEN*^HemDel^, *PTEN*^HomDel^) and further analyzed regarding to the *c-Jun* expression status by using the cBioPortal^18,19^. The data reveal a significant downregulation of *c-Jun* in patients with *PTEN*^HomDel^ and an almost unchanged *c-Jun* expression pattern in those with *PTEN*^HemDel^ compared to *PTEN*^WT^ patients, supporting our findings (Fig. 5g).

### High *c-Jun* expression levels and loss of *PTEN* are linked to poor prognosis in melanoma patients

**Fig. 6:**
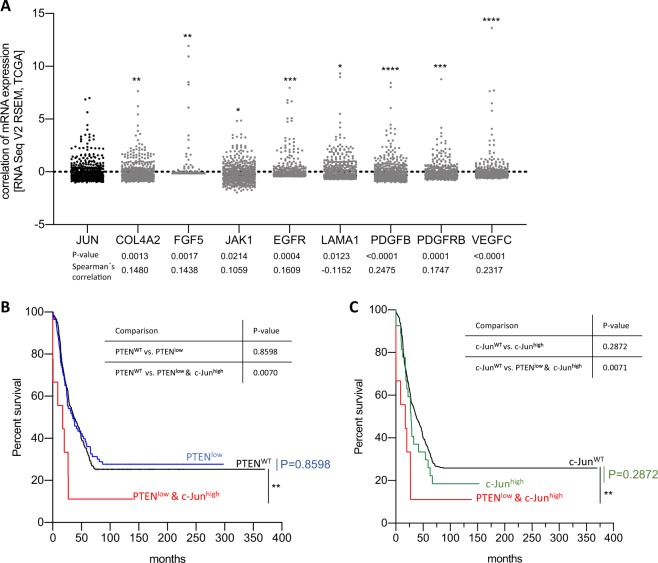
High c-Jun expression levels and loss of PTEN are linked to poor prognosis in melanoma patients. **a** mRNA expression (RNA-Seq) of *c-Jun* and *PI3K/AKT-*signaling members show a significant correlation in skin cutaneous melanoma patients (TCGA, Project ID: TCGA-SKCM). Spearman’s correlation is shown for each comparison. The scatter plots show the log2(*x* + 1) transformed RSEM normalized read counts of each melanoma patient (****P < 0.0001; ***P < 0.001; **P < 0.01; *P < 0.5). **b**, **c** Survival rates of the 471 skin cutaneous melanoma patients (TCGA, Project ID: TCGA-SKCM) depicted by PTEN and/or c-Jun expression. Comparison of the survival curves is illustrated in tables (**P < 0.001, Log-rank (Mantel–Cox) test)

Further analysis of the mRNA expression (RNA-Seq) showed a significant correlation of the expression of *c-Jun* and of *PI3K/AKT*-signaling members in skin cutaneous melanoma patients (TCGA, Project ID: TCGA-SKCM) confirming our findings (Fig. 6a). Further, we investigated overall survival of 471 skin cutaneous melanoma patients based on the *PTEN* and *c-Jun* expression status, and could clearly show that melanoma patients with alterations in both, *c-Jun* and *PTEN*, belong to the high risk group, whereas patients with either *PTEN*^WT^ or *c-Jun*^WT^ belong to the low risk group (Fig. 6b, c).

In summary, we identified a new, functional relevant relationship between the oncogenic transcription factor *c-Jun* and the tumor suppressor *PTEN* in malignant melanoma and a mechanism explaining the reduction in apoptosis and induction of tumor cell growth in *PTEN*^+^ melanoma cells, thus enabling the development of a malignant phenotype.

## Discussion

In previous studies, we demonstrated that the transcription factor *c-Jun* plays a critical role in the development and progression of malignant melanoma^3,5,9,10^. However, the detailed molecular mechanism of *c-Jun*’s influence on melanoma progression and development remains elusive given that only a subset of target genes is known. Previous studies have demonstrated that cancer-relevant genes, such as *cyclin D1*, *p53*, and *INK4A*^20,21^, are regulated by *c-Jun*. Moreover, we demonstrate that tumor-relevant genes, such as *FosB*, *WEE1*, *PVR*, *MAP1LC3B*, and *LGALS3*, are deregulated in melanoma by direct regulation via *c-Jun*^5^.

In this study, we investigated the detailed molecular role of the transcription factor *c-Jun* in melanoma cell lines representing different tumor stages and mutation status as determined by RNA-Seq and ChIP-Seq experiments. Differential gene expression in melanoma compared to NHEMs differs according to melanoma stage and mutation status of the sequenced melanoma cell lines, indicating various possible mechanisms leading to a malignant phenotype. Consistent with our findings, Haqq and colleagues^22^ also discovered gene expression differences between radial growth phase (RGP) and vertical growth phase (VGP) melanoma cells. It is well established that critical tumor-relevant signaling pathways, such as MAPK and PI3K, are deregulated in melanoma cells, leading to a high proliferative, migratory, and invasive potential of these cells and overcoming apoptosis^23,24^. Although oncogenic key players, such as *BRAF*, *NRAS*, *p53*, and *PTEN*, within these pathways are well characterized, the detailed molecular mechanisms remain elusive.

Our analysis revealed that *c-Jun* expression status correlates with *PTEN* expression in melanoma cells, indicating a dependency of *PTEN* and *c-Jun* to allow the development of a malignant phenotype. Computational clustering of our sequencing data suggested a dependency of *c-Jun* activity in melanoma cells based on the presence of the tumor suppressor *PTEN*. Moreover, the number of *c-Jun* peaks decreased as *PTEN* expression decreased and was ultimately lost. Mutations in members of the PI3K pathway have been extensively studied in many cancer entities. In melanoma, *PTEN* inactivation could be identified as the crucial step activating the PI3K pathway^25–27^. Consistently, recent studies also demonstrate a correlation between *c-Jun* and *PTEN* in nonmelanoma cancer. Hettinger and colleagues identified a direct downregulation of *PTEN* by *c-Jun*, leading to an upregulation of the *AKT* survival pathway in several cancer cells, such as pancreatic cells, glioblastoma cells, and transformed kidney epithelial cells^28^. Moreover, these results could also be confirmed in lung cancer cells^13^. However, the described *PTEN* and *c-Jun* expression pattern and binding site of *c-Jun* by Hettinger et al. was not revealed in our data, suggesting a different molecular relation between *PTEN* and *c-Jun* in melanoma.

Our data clearly revealed a functional correlation of *c-Jun* and *PTEN* in melanoma cells. In accordance with this finding, recent studies identified a crosstalk mechanism, whereby constitutive ERK activity suppresses *PTEN* in melanoma models and is mediated by modulation of *c-Jun*^29^. Furthermore, crosstalk between JNK and *PTEN* and thus the PI3K/AKT pathway could be detected in invasive adenocarcinoma of the prostate^30^. Interestingly, a mechanism exists that links ERK to JNK signaling in human melanoma. Hyperactivated ERK increases the stability of *c-Jun* protein and induces *c-Jun* transcription by CREB activation. C-Jun subsequently induces RACK1 transcription, which enhances JNK activation. Consequently, *c-Jun* is further stabilized and activated by JNK^31^.

Taken together, both identified crosstalk mechanisms support the hypothesis that *c-Jun* upregulation is the main link between the ERK/JNK-signaling pathways and the PI3K pathway, to overcome the tumor suppressive effect of *PTEN*, which would otherwise counteract the transformation process by inducing apoptosis.

Functional annotation analyses via multiple tools confirmed our findings. Analyses of the identified differentially expressed genes of melanoma cells resulted in a *PTEN*-independent enrichment of PI3K/AKT-signaling targets. Interestingly, we detected no increased *AKT* activity in *PTEN*-expressing melanoma cells and thus investigated if *c-Jun* regulates PI3K/AKT-related genes to promote malignancy. Indeed, our data revealed direct regulation of PI3K/AKT targets via *c-Jun* but not *AKT* itself, suggesting an overruling effect of *c-Jun* in the presence of *PTEN* during early melanoma development.

Previous studies described that increased levels of *c-Jun* in melanoma cell lines coincide with upregulation of phosphoinositide-dependent kinase 1 (PDK1) and phosphorylation of protein kinase C (PKC) and *AKT*^32^. Although, prior studies reported that *c-Jun* is a negative regulator inhibiting *PTEN*, detailed mechanisms by which *c-Jun* inhibits *PTEN* and thus indirectly increases *AKT* activity remain to be established^33,34^. Our data clearly showed no detectable alterations in *AKT* expression and activity in *c-Jun* and *PTEN-*coexpressing melanoma cells. Thus, *c-Jun* seems to play a crucial role in the presence of *PTEN* to enable the development of a malignant phenotype, which could be confirmed by various knockdown experiments. Previous data from Chen and colleagues support our results, revealing that *PTEN* inactivation results in cellular senescence in mouse embryonic fibroblasts^35^. Given that melanomas preferentially activate the PI3K pathway through inactivation of *PTEN*, it can be hypothesized that *c-Jun* expression in early melanoma stages protects melanoma cells with decreasing *PTEN* levels from this growth arrest.

Taken together, our study confirms a crucial role of the transcription factor *c-Jun* in melanoma development and progression in *PTEN*^+^ melanoma cells to overcome apoptosis and promote malignancy not only in vitro but also in vivo. TCGA data analysis by cBioPortal confirm and support our latest findings. Thus, the detection of *c-Jun* and *PTEN* coexpression in melanoma represents a promising diagnostic marker for highly aggressive melanoma cells with the potential to result in MET with the loss of both *c-Jun* and *PTEN*.

Further investigations will be necessary to determine the detailed tumor-promoting consequences of *c-Jun* activity in malignant melanoma.

## Material and methods

### Cell culture

Human melanoma cell lines Sbcl-2, WM3211, WM1366, WM793, WM1158, and WM9 (a generous gift from Dr. M. Herlyn, Wistar Institute, Philadelphia, USA) derived from RGP (Sbcl-2), VGP (WM3211, WM1366, WM793), and melanoma MET (WM1158, WM9) were maintained in a culture medium consisting of MCDB153 (Sigma-Aldrich, Steinheim, Germany) with 20% Leibovitz’s L-15 (PAA Laboratories, Coelbe, Germany), 2% FCS, 1.68 mM CaCl_2_ (Sigma), and 5 µg/ml insulin (Sigma-Aldrich, Steinheim, Germany) at 37 °C and 5% CO_2_. NHEM were derived from neonatal foreskin (NHEMs, PromoCell, Heidelberg, Germany) and were cultured in melanocyte growth media M2 at 37 °C and 5% CO_2_.

### Western blotting

Western blot analysis was performed as described previously^5^ using one of the following antibodies: anti-*c-Jun* (1 in 1000 dilution; Cell Signaling, Frankfurt am Main, Germany), anti-GAPDH (1 in 1000 dilution; Cell Signaling), anti ß-Actin (1 in 3000 dilution; Sigma Aldrich), anti-*PTEN* (1 in 40 dilution; Santa Cruz), anti-*AKT* (1 in 2000 dilution; Cell Signaling, Frankfurt am Main, Germany), and anti-P-*AKT* (1 in 2000 dilution; Cell Signaling, Frankfurt am Main, Germany). After three washes with TBS-T, the membrane was incubated for 1 h with an alkaline phosphate-coupled secondary anti-mouse (1 in 3000 dilution in TBS-T) or anti-rabbit (1 in 3000 dilution in TBS-T) IgG antibody (Chemicon, Hofheim, Germany), respectively HRP-coupled antibodies and then washed thrice in TBS-T. Finally, immunoreactions were visualized by NBT/BCIP (Sigma-Aldrich) staining or Clarity ^TM^ Western ECL Substrate (Biorad).

### siRNA transfection experiments with siRNA-Pools

siRNA transfection of WM1366 (*PTEN*^+^/*c-Jun*^+^) and WM9 (*PTEN*^−^/*c-Jun*^+^) melanoma cells was performed using the reverse transfection protocol of the Lipofectamine RNAiMAX reagent (Invitrogen, Carlsbad, CA, USA) according to the manufacturer´s instructions using siRNA-Pools as described previously^36^. For transfection experiments, 2 × 10^5^ cells were seeded each well in six-well plates. We applied “si-POOL-*AKT*”, “si-POOL-*PTEN*”, and “si-POOL-*c-Jun*” (functionally verified, by siTOOLs Biotech GmbH, Planegg, Germany) for specific knockdown of *AKT1*, *AKT2*, *AKT3*, *PTEN*, and *c-Jun*, respectively.

### Analysis of gene expression by quantitative PCR

cDNAs of total RNA fractions were generated using Super-Script II Reverse Transcriptase Kit (Invitrogen, Groningen, The Netherlands). qRT-PCR was performed on a LightCycler (Roche, Mannheim, Germany) as described previously^5^. Annealing and melting temperatures were optimized for each primer set (Table 2). ß-Actin was used for normalization.

**Table 2:** Oligonucleotide sequences and qRT–PCR conditions

Gene	Primer sequences (fwd/rev)	*T*_a_ (°C)	*T*_M_ (°C)
*ß-actin*	5′-CTACGTCGCCCTGGACTTCGAGC-3′	60–68	80
	5′-GATGGAGCCGCCGATCCACACGG-3′		
*c-Jun*	5′-TTCCTCCCGTCCGAGAGCGG-3′	60–70	80
	5′-TCGGCGTGGTGGTGATGTGC-3′		
*AKT1*	5′-AGCCCACCCTTCAAGCCCCA-3′	60	82
	5′-CTGCGCTCGCTGTCCACACA-3′		
*AKT3*	5′-TCTGCCTTGGACTATCTACA-3′	60	76
	5′-AATTTTTATGTGGCCATCT-3′		
*PTEN*	5′-TGTGGTCTGCCAGCTAAAGG-3′	60	80
	5′-AGGTTTCCTCTGGTCCTGGT-3′		

### RNA-Seq library preparation and mapping

RNA-Seq samples and libraries were prepared as described previously^37^. Library preparation was performed with at least two biological replicates. Sequencing was performed according to the paired-end RNA sequencing protocols from Illumina on a HiSeq2000 with paired-end module (Illumina, Inc.). Fifty samples were sequenced from each side of a fragment ~100 bp long with an average number 20 million reads per sample.

Paired-end reads were aligned to the human reference genome sequence (hg38) using the STAR alignment software (v 2.5.2a)^38^. Only reads that mapped to a single unique location were considered for further analysis. Counts for RNA-Seq reads were calculated using the feature-counts software (v 1.4.6-p5).

### Chromatin immunoprecipitation

ChIP was performed with the human melanoma cell lines Sbcl-2, WM3211, WM1366, WM793, WM1158, WM9 and NHEM that had not exceeded six passages. In terms of ChIP experiments with transcription factors, we performed double crosslinking with disuccinimidyl glutarate (DSG, Thermo Scientific, Rockford, USA) before a fixation step with formaldehyde. Chromatin from 10 × 10^6^ cells of each cell line was crosslinked in 1/10th volume of fixation buffer (50 mM HEPES/KOH, pH 7.9, 11% formaldehyde) for 10 min at room temperature and quenched by 0.125 M glycine. After two washes with PBS and PMSF, the cells were scraped and centrifuged at 4 °C and 3500×*g* for 10 min. The supernatant was discarded, and the pellet was resuspended in 15 ml of lysis buffer 1 (5 mM PIPES [pH 8.0], 85 mM KCl, 0.5% NP-40, 1x Roche Complete, EDTA-free protease inhibitor) and incubated for 10 min on ice. After centrifugation of 5 min at 4 °C and 3500×*g*, the supernatant was discarded, and the pellet resuspended in 15 ml lysis buffer 2 (50 mM Tris–HCl, pH 8.0, 10 mM EDTA, 1% SDS, 1× Roche Complete, EDTA-free protease inhibitor). The suspension was then incubated on ice for an additional 10 min and was examined under the microscope for quality assessment of nuclear prep. The nuclei were pelleted at 4 °C and 3500×*g* for 5 min. The supernatant was discarded, and the pellet was resuspended in the desired volume of sonication buffer (10 × 10^6^ cells in 450 µl sonication buffer: 16.7 mM Tris–HCl [pH 8.0], 1.2 mM EDTA, 167 mM NaCl, 0.01% SDS, 1.1% Triton X-100, 1× Roche Complete, EDTA-free protease inhibitor). Cross-linked chromatin was sheared to an average DNA fragment size of ~400–600 bp using a Branson Sonifier 250 (Danbury, CT). After centrifugation, 2% of supernatant was used as input. After preclearing with Sepharose CL-4B beads (Sigma) for 2 h, chromatin samples from 10 × 10^6^ cells were immunoprecipitated overnight with 5 μg of rabbit polyclonal antibody anti-*c-Jun* (Santa Cruz Biotechnology Inc.) or anti- H3K37ac (Abcam). Immunocomplexes were recovered by 3 h of incubation with protein A-Sepharose beads (GE Healthcare) at 4 °C. These beads were washed thrice and resuspended in 1 mM EDTA and 10 mM Tris–HCl, pH 8.1. Precipitates were serially washed with 400 μl Washing Buffer I (2 mM EDTA, 20 mM Tris–HCl [pH 8.0], 0.1% SDS, 1% Triton X-100, 150 mM NaCl), Washing Buffer III (1 mM EDTA, 10 mM Tris–HCl [pH 8.0], 1% NP-40, 1% Deoxycholate, 0.25 M LiCl) and thrice with 1 mM EDTA and 10 mM Tris–HCl, pH 8.0. Precipitated chromatin complexes were removed from the beads through a 20-min incubation with 100 μl of 1% SDS, 0.1 M NaHCO_3_ with vortexing each 5 min. This step was repeated twice with 10-min incubation times. After reverse cross-linking, DNA was purified using the QIAquick PCR purification kit (Qiagen).

### ChIP-seq and mapping

DNA from ChIP (10–50 ng) was adapter ligated and polymerase chain reaction amplified according to the manufacturer’s instructions (Illumina, San Diego, USA). ChIP fragments were sequenced for 36 cycles on Illumina Genome Analyzer according to the manufacturer´s protocol. Sequence tags of all experiments were mapped to the current human reference sequence (GRCh8/hg38) using Bowtie 2 (v 2.2.7)^39^, and only uniquely mapped tags were used for downstream analysis. Tag counts were normalized to 10^7^ specifically mapped tags.

### Sequencing data analysis

The raw RNA-Seq counts were used for differential gene expression analysis that was performed using DESeq2 (v 1.14.1)^40^. Differentially expressed genes with a false-discovery rate (FDR) <0.05 were regarded as statistically significant. Normalization was performed by library size based on the raw counts.

Genome Ontology annotation and RNA-Seq reads annotation were performed using scripts provided by STAR (based on GENCODE V24).

Analysis of mapped ChIP-seq tags was performed using HOMER, which is freely available at http://biowhat.ucsd.edu/homer/^41^. The ChIP-Seq sequence data were assessed for abnormal GC content, excessive clonal amplification (multiple tags starting at the same genomic position), inherent sequence bias in the sequenced tags and surrounding genomic positions and contamination with plasmid/cDNA sequences. The identification of ChIP-Seq peaks (bound regions) was performed using a custom approach (HOMER) that combines features of previously published methods. Peaks were defined at a 0.001 estimated false discovery rate. Genome Ontology annotation and ChIP-Seq tag annotation of peak sets was performed using scripts provided by HOMER (based on GENCODE V24). Next generation sequencing data used in this study are listed below:

**Table:** 

ChIP-seq			
Cell type	Target protein	Total uniquely mapped tags (hg38)	Total peaks identified
NHEM	H3K27ac	22,173,094	72,031
Sbcl-2	*c-Jun*	8,977,157	54,943
Sbcl-2	H3K27ac	16,073,130	66,130
WM3211	*c-Jun*	14,381,672	91,277
WM3211	H3K27ac	29,299,544	87,743
WM1366	*c-Jun*	14,015,631	28,061
WM1366	H3K27ac	16,732,104	71,798
WM793	*c-Jun*	12,148,216	29,726
WM793	H3K27ac	21,459,505	79,942
WM1158	*c-Jun*	15,632,274	28,000
WM1158	H3K27ac	17,825,517	78,432
WM9	*c-Jun*	13,634,991	1164
WM9	H3K27ac	18,466,555	73,667

RNA-Seq	
Cell type	Total mapped read counts (hg38)
NHEM_rep1	10,306,403
NHEM_rep2	15,287,883
Sbcl-2_rep1	9,710,494
Sbcl-2_rep2	9,217,901
WM3211_rep1	6,644,449
WM3211_rep2	8,660,603
WM1366_rep1	5,447,622
WM1366_rep2	11,600,401
WM793_rep1	9,590,158
WM793_rep2	9,065,432
WM1158_rep1	12,014,897
WM1158_rep2	6,173,661
WM9_rep1	15,814,898
WM9_rep2	3,534,649

### Attachment assay using the xCELLigence system

Attachment was determined using the xCELLigence System of Roche Diagnostics (Penzberg, Germany) as described previously^42^. This instrument monitors the behavior of the cells in real time by measuring the electrical impedance across interdigitated microelectrodes covering the bottom of ‘E-plates’. Electrode impedance is displayed as cell index values.

### Cell proliferation assay

Melanoma cells (4000 cells/well) were seeded in a 96-well plate, the medium was aspirated and cells were incubated with the dye-binding solution from the CyQUANT^®^ NF Cell Proliferation Assay Kit (Invitrogen) according to the manufacturer’s protocol. Finally, fluorescence was measured in an EMax Microplate Reader (MWG Biotech, Ebersberg, Germany) after 1 and 72 h.

### Migration assay

Migration potential of melanoma cells was quantified using the Cultrex 96-Well Cell Migration Assay (Trevigen, Gaithersburg, USA) according to the manufacturer’s instructions as described previously^43^. Briefly, melanoma cells were seeded into the upper compartment of the provided 96-well plate (20,000 cells/well) in RPMI. The lower compartment was filled with RPMI supplemented with conditioned medium from fibroblasts and 20% (directed migration) or 0% (undirected migration) FCS, respectively, as chemoattractants. After incubation at 37 °C for 4 h cell migration was quantified by fluorimetry with an EMax Microplate Reader (MWG Biotech, Ebersberg, Germany).

### GO analysis

The Database for Annotation, Visualization, and Integrated Discovery (DAVID; v 6.8)^44,45^ was used to investigate biological meaning of the obtained differential expressed gene lists by DESeq2 of our RNA-Seq data and also GO terms for differentially expressed genes potentially regulated by *c-Jun*. The STRING database (v 10.5)^46,47^ was used to visualize predicted associations of differentially expressed genes or differentially expressed genes potentially regulated by *c-Jun*, respectively. Moreover, enriched GO terms of different gene sets were also identified by STRING.

Gene Set enrichment analyses were performed using GSEA (v 2.0)^48,49^.

GREAT^50,51^ was used to identify GO terms of non-coding genomic regions (ChIP-Seq peaks) by analyzing the annotations of the nearby genes. The association rule was set as follows: proximal, 10 kb upstream and 10 kb downstream (any gene in this interval relative to input regions is included); plus distal, up to 1000 kb (if no gene is present in the proximal interval, the closest gene in this distal interval is included).

### Statistical analysis

In the bar graphs, results are expressed as mean ± s.d. (range). Comparisons between qRT-PCR data were made using the Student’s unpaired *t*-test. A *P*-value of <0.05 was considered as statistically significant (NS: not significant, *P < 0.05, **P < 0.01, ***P < 0.001, ****P < 0.0001). Group comparisons of Next Generation Sequencing data were made by the non-parametric Wilcoxon test and of TCGA patient data by the Welch's *t*-test after removing identified outliers. All calculations were performed using the GraphPad Prism Software (V8, GraphPad Software, Inc., San Diego, CA, USA) or RStudio (Version: 1.1.456, RStudio, Inc., Boston, MA, URL, http://www.rstudio.com).

## Supplementary information

suppl. figures
