# Supplementary material for: C-Jun drives melanoma progression in PTEN wild type melanoma cells
Source: Cell Death Dis. 2019 Aug 5;10(8):584. doi: 10.1038/s41419-019-1821-9 (PMC6680049; doi:10.1038/s41419-019-1821-9)
Supplement: Supplementary file 1 — suppl. figures [file 41419_2019_1821_MOESM1_ESM.docx]

**Supplementary data for**

***c-Jun* drives melanoma progression in *PTEN* wild type melanoma cells**

M. Kappelmann^1,2^, C. Gebhard^3,4^, A. O. Matthies^1^, S.Kuphal^1^, M. Rehli^3,4^, A. K. Bosserhoff^1,5,§^

*^1^Institute of Biochemistry (Emil-Fischer Center), Friedrich-Alexander University Erlangen-Nürnberg, Erlangen, Germany*

*^2^ Faculty of Applied Health Care Sciences, University of Applied Science Deggendorf, Germany*

*^3^Department of Internal Medicine III, University Hospital Regensburg, Regensburg, Germany*

*^4^Regensburg Center for Interventional Immunology (RCI), c/o University Hospital Regensburg, Regensburg, Germany.*

*^5^Comprehensive Cancer Center (CCC)-EMN, Erlangen, Germany*

| **Cell line** | **Cell Stage** | **BRAF** | **PTEN Mutation** | **NRAS** | **P53** | **CDK4** |
| --- | --- | --- | --- | --- | --- | --- |
| NHEM |  | WT | WT |  |  |  |
| Sbcl2 | PT (RGP) | WT | WT | Q61K | WT | WT |
| WM3211 | PT (VGP) | WT | WT | WT | Mut | WT |
| WM1366 | PT (VGP) | WT | WT | Q61L | WT | WT |
| WM793 | PT (VGP) | V600E | Hemizygous deletion  (Mut W274X, Ex 8) | WT | WT | K22Q |
| WM1158 | MET | V600E | Hemizygous deletion  (Mut V343E) | WT | WT | WT |
| WM9 | MET | V600E | Homozygous deletion  (Ex 3-9) | WT | WT | WT |

**Supplement Table 1**

**Cell line characteristics**

| **c-Jun Peak Sets** |  |
| --- | --- |
| **Set 1** | Common c-Jun peaks of all melanoma cells |
| **Set 2** | Common c-Jun peaks of all PT cells |
| **Set 3** | Common c-Jun peaks of all MET cells |
| **Set 4** | Common c-Jun peaks of all PTEN^WT^ cells |
| **Set 5** | Common c-Jun peaks of all PTEN^HemDel^ cells |

**Supplement Table 2**

**Generated ChIP-Seq c-Jun peak sets.**

| **comparison** | ***total diffgenes*** | **c-Jun peaks** | ***diffgenes Jun*** |
| --- | --- | --- | --- |
| NHEM-PTEN^WT^ | 4188 | common in PTEN^WT^ | 1084 |
| NHEM-PTEN^HemDel^ | 2764 | common in PTEN^HemDel^ | 736 |
| NHEM-PTEN^HomDel^ | 2241 |  |  |

**Supplement Table 3**

**Amount of *total diffgenes* and *diffgenes Jun* of all generated gene sets.**

**Supplement Table 4**

**Differentially expressed PI3K/AKT members in PTEN^WT^, PTEN^HemDel^ and PTEN^HomDel^ and differentially expressed PI3K/AKT members in PTEN^WT^, PTEN^HemDel^ regulated by c-Jun.**

| **differentially expressed PI3K/AKT members** | | | | |
| --- | --- | --- | --- | --- |
| **in PTEN^WT^** | **in PTEN^WT^ regulated by** c-Jun | **in PTEN^HemDel^** | **in PTEN^HemDel^ regulated by** c-Jun | **in PTEN^HomDel^** |
| CDKN1A | COL4A2 | CDK6 | ITGA3 | CDK6 |
| COL4A6 | FGF5 | FGF1 | LAMC2 | JAK1 |
| EGFR | ITGA3 | FGF13 | KIT | KIT |
| FGF1 | JAK1 | FGF2 | FGF2 | LAMA1 |
| ITGA2 | LAMC2 | KIT | PDGFRB | VEGFC |
| ITGA3 | MAPK1 | LAMA1 | PRKCA | CCND1 |
| CCND1 | CCND1 | BCL2 | VEGFC | PGF |
| COL4A1 | EGFR | COL4A6 | LAMA2 | PIK3CD |
| COL4A2 | FGF2 | EGFR | BCL2 | SOS2 |
| FGF2 | KIT | FGF5 | CDK6 | VEGFB |
| FGF5 | KITLG | FN1 |  | PTEN |
| FN1 | LAMA1 | IGF1R |  | FGF13 |
| GRB2 | LAMA5 | IL6 |  | RAF1 |
| IGF1R | LAMB3 | ITGA3 |  | FGFR1 |
| JAK1 | PDGFB | LAMA2 |  |  |
| KIT | PDGFRB | LAMA5 |  |  |
| KITLG | PRKCA | LAMB3 |  |  |
| LAMA1 | VEGFC | LAMC2 |  |  |
| LAMA5 |  | MAP2K2 |  |  |
| LAMB3 |  | PDGFA |  |  |
| LAMC2 |  | PDGFB |  |  |
| MAP2K2 |  | PDGFRB |  |  |
| MAPK1 |  | PRKCA |  |  |
| NRAS |  | PTEN |  |  |
| PDGFA |  | SOS2 |  |  |
| PDGFB |  | VEGFA |  |  |
| PDGFRB |  | VEGFB |  |  |
| PGF |  | VEGFC |  |  |
| PIK3CD |  |  |  |  |
| PIK3R3 |  |  |  |  |
| PRKCA |  |  |  |  |
| SOS2 |  |  |  |  |
| VEGFA |  |  |  |  |
| VEGFB |  |  |  |  |
| VEGFC |  |  |  |  |

**Supplement Table 5**

Detailed previously described functions in melanoma of each differentially expressed gene regulated by c-Jun.

| **c-Jun regulated PI3K/AKT signaling members in PTEN^WT^** | | |
| --- | --- | --- |
| \| **Gene** \| **Description** \| **Function in melanoma** \| \| --- \| --- \| --- \| \| **LAMB3**  Laminin subunit beta 3 \| Influential role in cell differentiation, migration, and adhesion. \| Association with melanoma metastasis^1^. \| \| **EGFR**  Epidermal growth factor receptor \| Important role in promoting cell proliferation and cell survival. \| Pathogenesis of cancer^2^.  Important for growth, and contribution in migration and metastatic potential^3^. \| \| **FGF2**  Fibroblast growth factor 2 \| Binds heparin and possess broad mitogenic and angiogenic activities.  Plays important roles in diverse biological processes, such as limb and nervous system development, wound healing, and tumor growth. \| Regulates endothelial and melanoma cell migration^4^.  Regulators of melanoma angiogenesis and metastasis^5^.  Modulates melanoma adhesion and migration through a syndecan-4-dependent mechanism^6^. \| \| **PDGFB**  Platelet derived growth factor subunit B \| plays a role in a wide range of developmental processes by activating PDGF receptor tyrosine kinases. \| Stimulate the development of tumor stroma and new blood vessels.  Function as an autocrine growth factor, as well as an angiogenesis factor, in cutaneous malignant melanoma (CMM) tumor development^7^. \| \| **LAMC2**  Laminin subunit gamma 2 \| Role in biological processes including cell adhesion, differentiation, migration, signaling, neurite outgrowth and metastasis. \| Association with melanoma metastasis^8^. \| \| **VEGFC**  Vascular endothelial growth factor C \| Promotes angiogenesis and endothelial cell growth, and can also affect the permeability of blood vessels. \| Promotes immune tolerance^9^.  Act as most potent lymphangiogenic growth factor^10^.  Influential role in the metastasis of malignant melanoma^11^. \| \| **PRKCA**  Protein kinase C alpha \| Plays roles in many different cellular processes, such as cell adhesion, cell transformation, cell cycle checkpoint, and cell volume control. \| Metastasis of malignant melanoma in patients^12^.  Enhanced G1 to S transition^13^.  Exponential proliferation of human melanoma cells has been associated with low levels of protein kinase C (PKC)-alpha^14^. \| \| **FGF5**  Fibroblast growth factor 5 \| Possess broad mitogenic and cell survival activities  Involved in a variety of biological processes, including embryonic development, cell growth, morphogenesis, tissue repair, tumor growth and invasion.  Also act as oncogene. \| Associated with the malignancy of melanoma cells^15^.  Oncogenic potential in melanoma cells and contributes to melanoma growth^16^. \| \| **ITGA3**  Integrin subunit alpha 3 \| Important roles in cell growth, proliferation, migration and apoptosis. \| Significant role in melanoma cell motility^17^. \| \| **PDGFRB**  Platelet derived growth factor receptor beta \| Important role in the normal development of the cardiovascular system.  Helps in the rearrangement of the actin cytoskeleton. \| Upregulation of EGFR and platelet-derived growth factor receptor-β (PDGFRB), which confer resistance to BRAF and MEK inhibitors^18^.  Co-expression of the growth factor and receptor suggests their role in autocrine or paracrine growth mechanisms in melanoma^19^. \| \| **LAMA5**  Laminin subunit alpha 5 \| Important roles in wide variety of biological processes including cell adhesion, differentiation, migration, signaling, neurite outgrowth and metastasis. \| Role in melanoma cell attachment and migration on extracellular matrix proteins^20^. \| \| **JAK1**  Janus kinase 1 \| Key role in interferon-alpha/beta and interferon-gamma signal transduction. \| Promotes BRAFi resistance in melanoma^21^.  Helps in the survival mechanism of malignant melanoma^22^. \| \| **MAPK1**  Mitogen-activated protein kinase 1 \| Act as an integration point for multiple biochemical signals, and are involved in a wide variety of cellular processes such as proliferation, differentiation, transcription regulation and development. \| Plays role in regulating survival of human melanoma cells upon endoplasmic reticulum stress through autophagy^23^. \| \| **COL4A2**  Collagen type IV alpha 2 chain \| Inhibitor of angiogenesis and tumor growth. \| Immunolocalization and distribution patterns of type IV collagen alpha chains are associated with the progression of OMM^24^. \| \| **CCND1**  Cyclin D1 \| Function as regulators of CDK kinases. It also plays an important role in cell cycle G1/S transition. \| Influence the metastatic progression, survival, and the localization of metastases^25^. \| \| **KIT**  Proto-oncogene receptor tyrosine kinase \| Encodes a protein, which is a type 3 transmembrane receptor for MGF (mast cell growth factor, also known as stem cell factor). \| KIT functions as an oncogene^26^.  Metastasis of melanoma^27^. \| \| **LAMA1**  Laminin subunit alpha1 \| Role in wide variety of biological processes including cell adhesion, differentiation, migration, signaling, neurite outgrowth and metastasis. \|  \| \| **KITLG**  KIT Ligand \| A pleiotropic factor that acts in utero in germ cell and neural cell development, and hematopoiesis, all believed to reflect a role in cell migration. \| Contribution to melanoma predisposition and tumorigenesis through gene-environment interactions^28^. \| \|  \|  \|  \| | | |
| **c-Jun regulated PI3K/AKT signaling members in PTEN^HemDel^** | | |
| **PDGFRB**  Platelet derived growth factor receptor beta | Important role in the normal development of the cardiovascular system.  Helps in the rearrangement of the actin cytoskeleton. | Upregulation of EGFR and platelet-derived growth factor receptor-β (PDGFRB), which confer resistance to BRAF and MEK inhibitors^18^.  Coexpression of the growth factor and receptor suggests their role in autocrine or paracrine growth mechanisms in melanoma^19^. |
| **ITGA3**  Integrin subunit alpha 3 | Important roles in cell growth, proliferation, migration and apoptosis. | Significant role in melanoma cell motility^29^. |
| **PRKCA**  Protein kinase C alpha | Plays roles in many different cellular processes, such as cell adhesion, cell transformation, cell cycle checkpoint, and cell volume control. | Metastasis of malignant melanoma in patients^12^.  Enhanced G1 to S transition^13^.  Plays an important role in melanoma cell migration^30^.  Exponential proliferation of human melanoma cells has been associated with low levels of protein kinase C (PKC)-alpha^31^. |
| **VEGFC**  Vascular endothelial growth factor C | Promotes angiogenesis and endothelial cell growth, and can also affect the permeability of blood vessels. | Promotes the ingrowth and invasion of lymphatics in many different tumor types, including melanoma^32^.  VEGF-C expression in primary cutaneous melanoma plays a role in the lymphatic spread of the tumor^33^. |
| **LAMC2**  Laminin subunit gamma 2 | Role in biological processes including cell adhesion, differentiation, migration, signaling, neurite outgrowth and metastasis. | Association with melanoma metastasis^8^. |
| **CDK6**  Cyclin dependent kinase 6 | Important role in cell cycle G1 phase progression and G1/S transition. | Regulation of melanoma G1-S cell cycle^34^. |
| **FGF2**  Fibroblast growth factor 2 | FGF family members bind heparin and possess broad mitogenic and angiogenic activities.  Significant roles in biological processes, such as limb and nervous system development, wound healing, and tumor growth. | Specifically regulates melanoma cell ability to migrate through a syndecan-4-dependent mechanism^6^.  Dysregulated expression of basic fibroblast growth factor [fibroblast growth factor 2 (FGF-2)] mediates autocrine growth of melanoma cells^35^.  Cooperation in the induction of increased Angiogenesis in human melanoma^36^. |
| **LAMA2**  Laminin subunit alpha 2 | Mediates the attachment, migration, and organization of cells into tissues during embryonic development by interacting with other extracellular matrix components. | Association with malignant transformation^37^.  Tumor progression in human malignant melanoma^38^. |
| **BCL2** | Encodes an integral outer mitochondrial membrane protein that blocks the apoptotic death of some cells such as lymphocytes. | Maintaining the cellular integrity in melanoma. Protecting melanoma cells from apoptosis and mediating other processes thus enhancing aggressive phenotype^39^. |
| **KIT**  Proto-oncogene receptor tyrosine kinase | Encodes a protein, which is a type 3 transmembrane receptor for MGF (mast cell growth factor, also known as stem cell factor). | KIT functions as an oncogene^26^. |


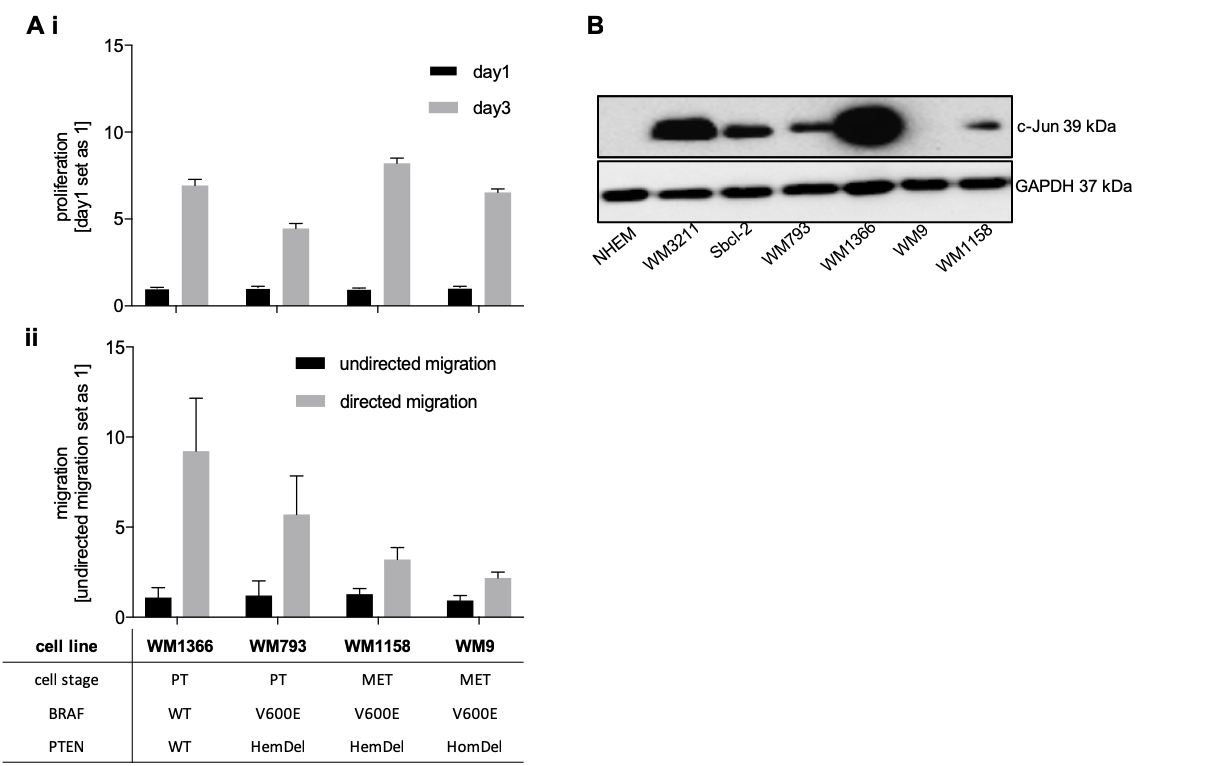


GAPDH-37 kDa

c-Jun-43 kDa

**Supplement Figure 1.**

**(A)** Functional assays showing the proliferative (i) and migratory potential (ii) of the melanoma cell lines WM1366, WM793, WM1158, WM9 in terms of their tumor phase and mutation status. **(B)** Western blot analysis of c-Jun expression status in the melanoma cell lines WM3211, Sbcl2, WM793, WM1366,WM9 and WM1158 compared to NHEMs. Original Western blot, GAPDH served as loading control.


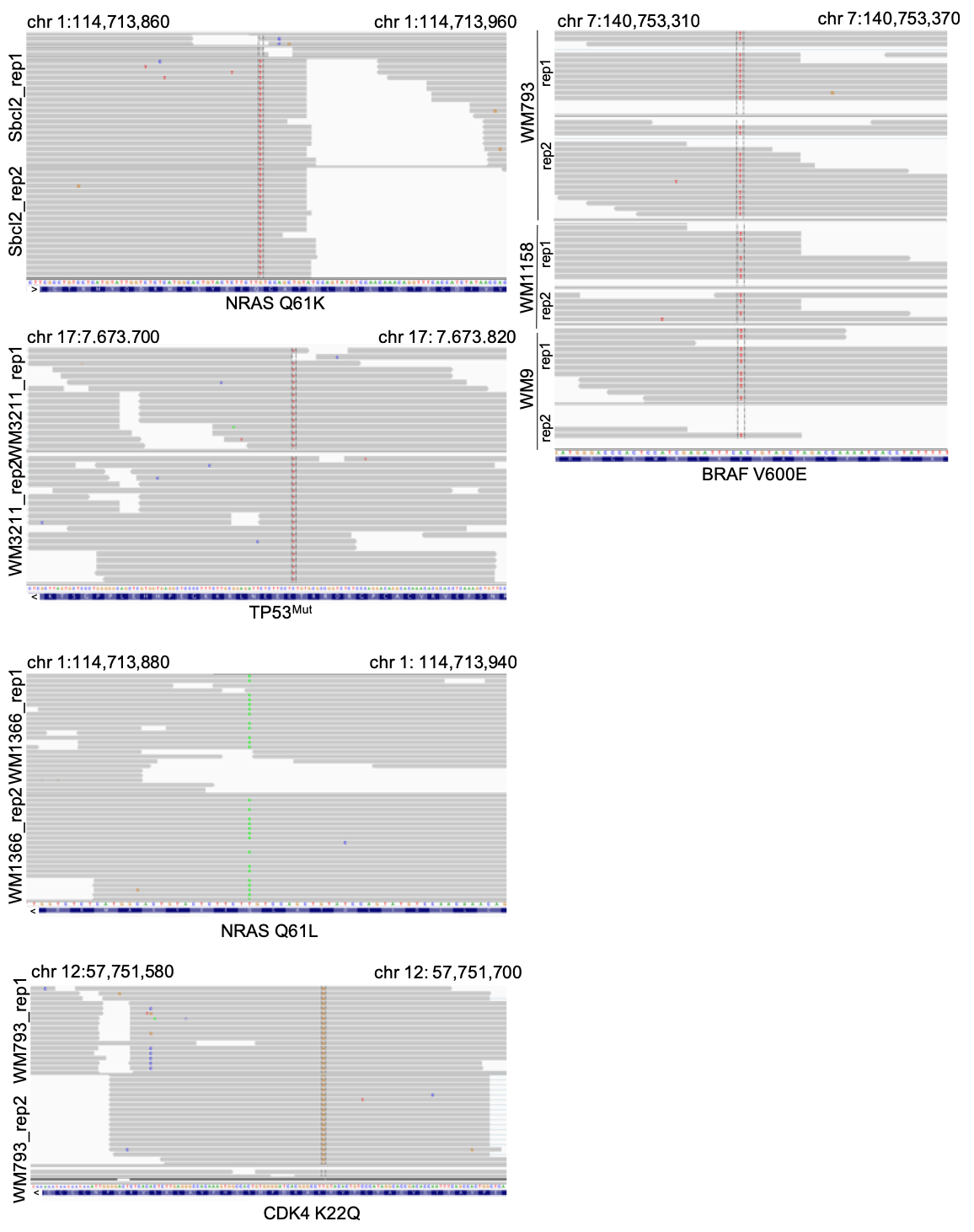


**Supplement Figure 2.** Verification of previously described mutations of the melanoma cell lines Sbcl2, WM3211, WM1366, WM793, WM1158 and WM9 by visualisation of the RNA sequencing data in the IGV browser.


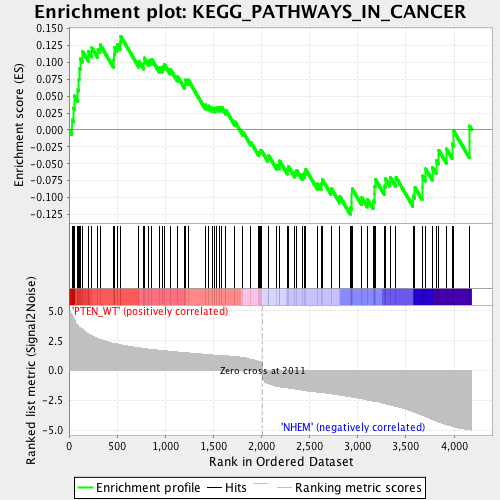
**
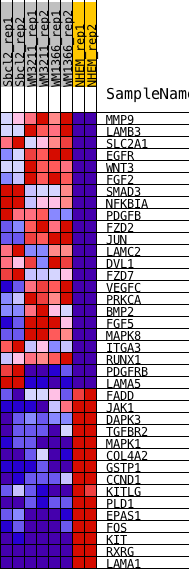
**
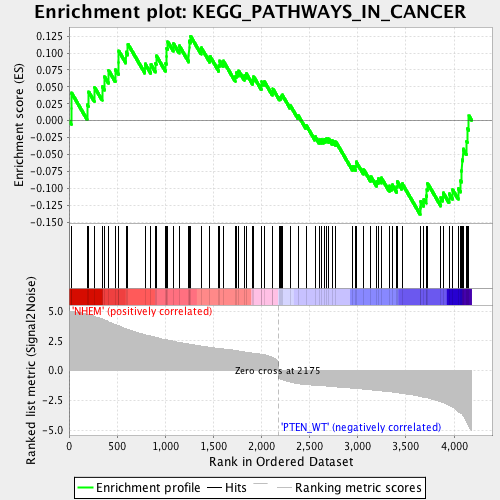

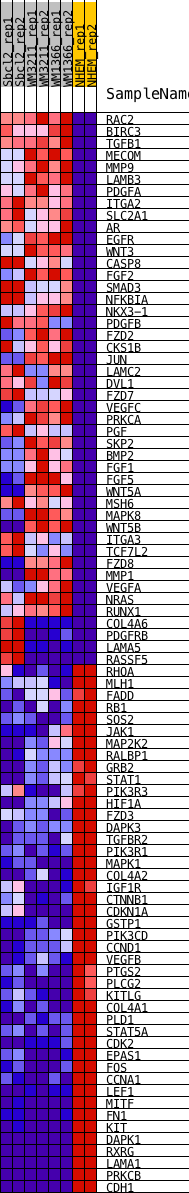

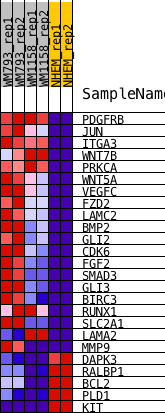

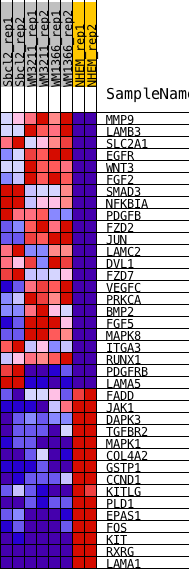


**A**

NHEM-PTEN^WT^_c-Jun

NHEM-PTEN^WT^

🡪

🡪

🡪

🡪

🡪

🡪

🡪

🡪

🡪

🡪

🡪

🡪

🡪

🡪

🡪

🡪 JUN regulated

PI3K-AKT members

🡪

🡪

🡪

🡪

🡪

🡪

🡪

🡪

🡪

🡪

🡪

🡪

🡪

🡪

🡪

🡪

🡪

🡪

🡪 JUN regulated

PI3K-AKT members

🡪

🡪

🡪

🡪 JUN regulated

PI3K-AKT members

🡪

🡪

🡪

🡪

🡪

🡪

🡪

🡪

🡪

🡪

🡪

🡪

🡪

🡪

🡪


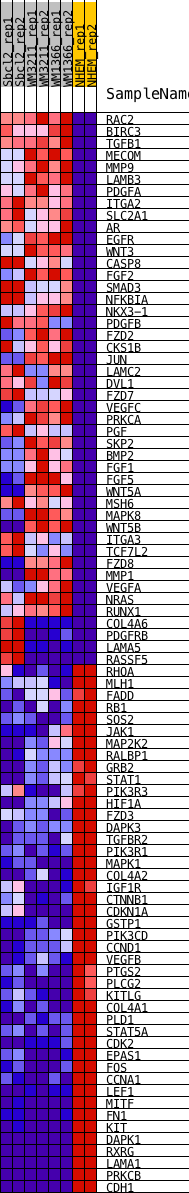


NHEM-PTEN^HemDel^_c-Jun

NHEM-PTEN^HemDel^

**
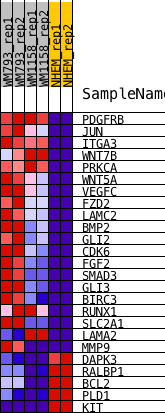
**
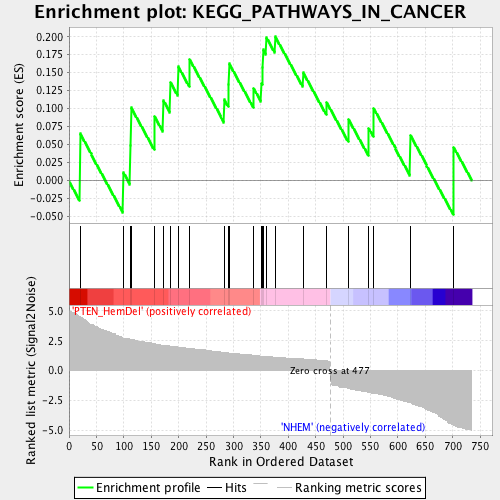

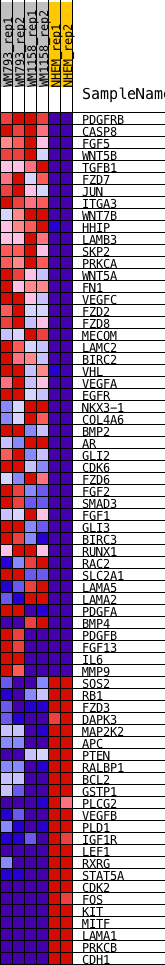

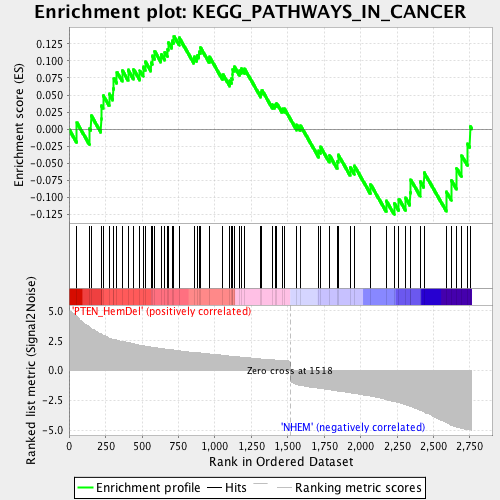

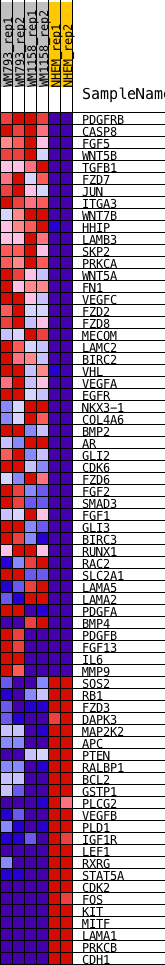


🡪 JUN regulated

PI3K-AKT members

🡪

🡪

🡪

🡪

🡪

🡪

🡪

🡪

🡪

NHEM-PTEN^HomDel^

**B**

**
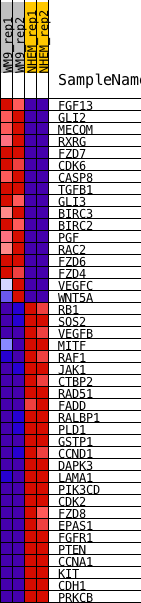
**
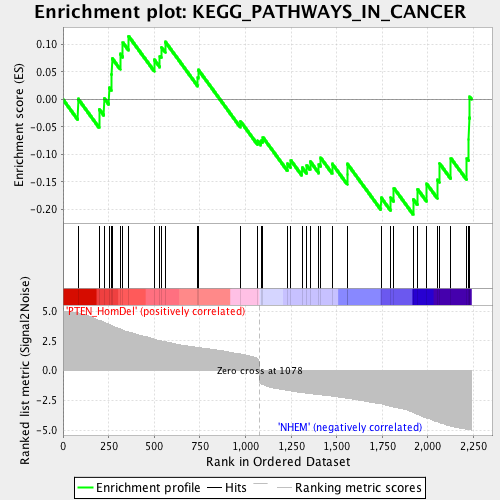


🡪

🡪 deregulated

PI3K-AKT members

🡪

🡪

🡪

🡪

🡪

🡪

🡪

🡪

**Supplement Figure 3. (A) GSEA results of the RNA-Seq data.** Enrichment plots and the corresponding *Blue-Pink O' Gram in the Space of the Analyzed GeneSets* of *Pathways in cancer* of all comparisons (NHEM versus PTEN^WT^, *PTEN^WT^c-Jun*, PTEN^HemDel^, *PTEN^HemDel^c-Jun* or PTEN^HomDel^) are shown. The outcome is represented in *Profile of the Running ES Score & Positions of GeneSet Members on the Rank Ordered List*. **(B) c-Jun target gene validation.** qRT-PCR of c-Jun knockdown compared to control transfected WM1366 (PTEN^WT^) melanoma cells show a significant upregulation of CCND1 after sic-Jun transfection and a significant downregulation of FGF5 and EGFR. The bars show the mean ±s.d. four independent experiments; (***P<0.001; **P<0.01; *P<0.5).


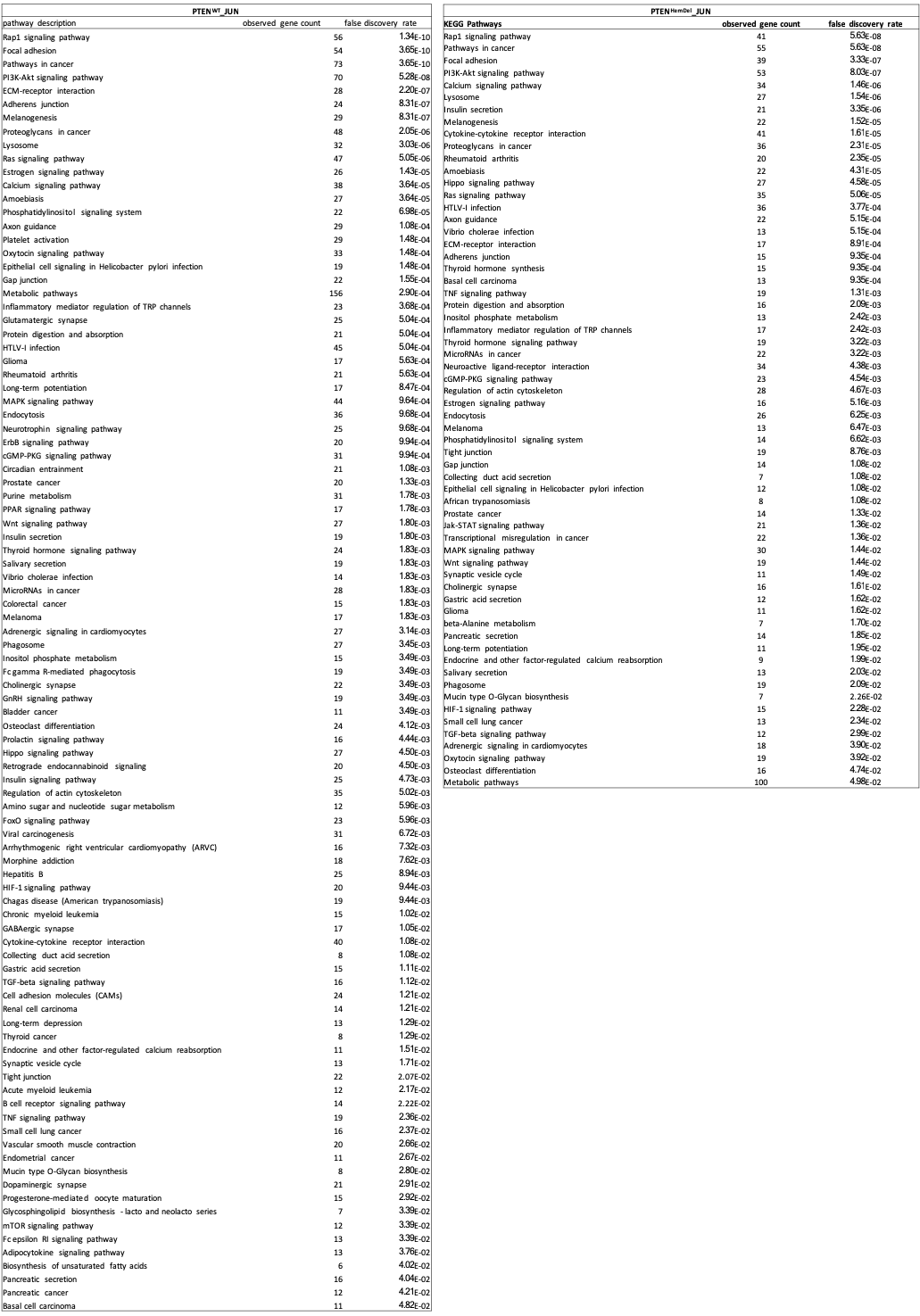


**Supplement Figure 4.** Functional analysis of ChIP-Seq cis regulatory regions (GREAT) combined with RNA-Seq differentially expression results via STRING.


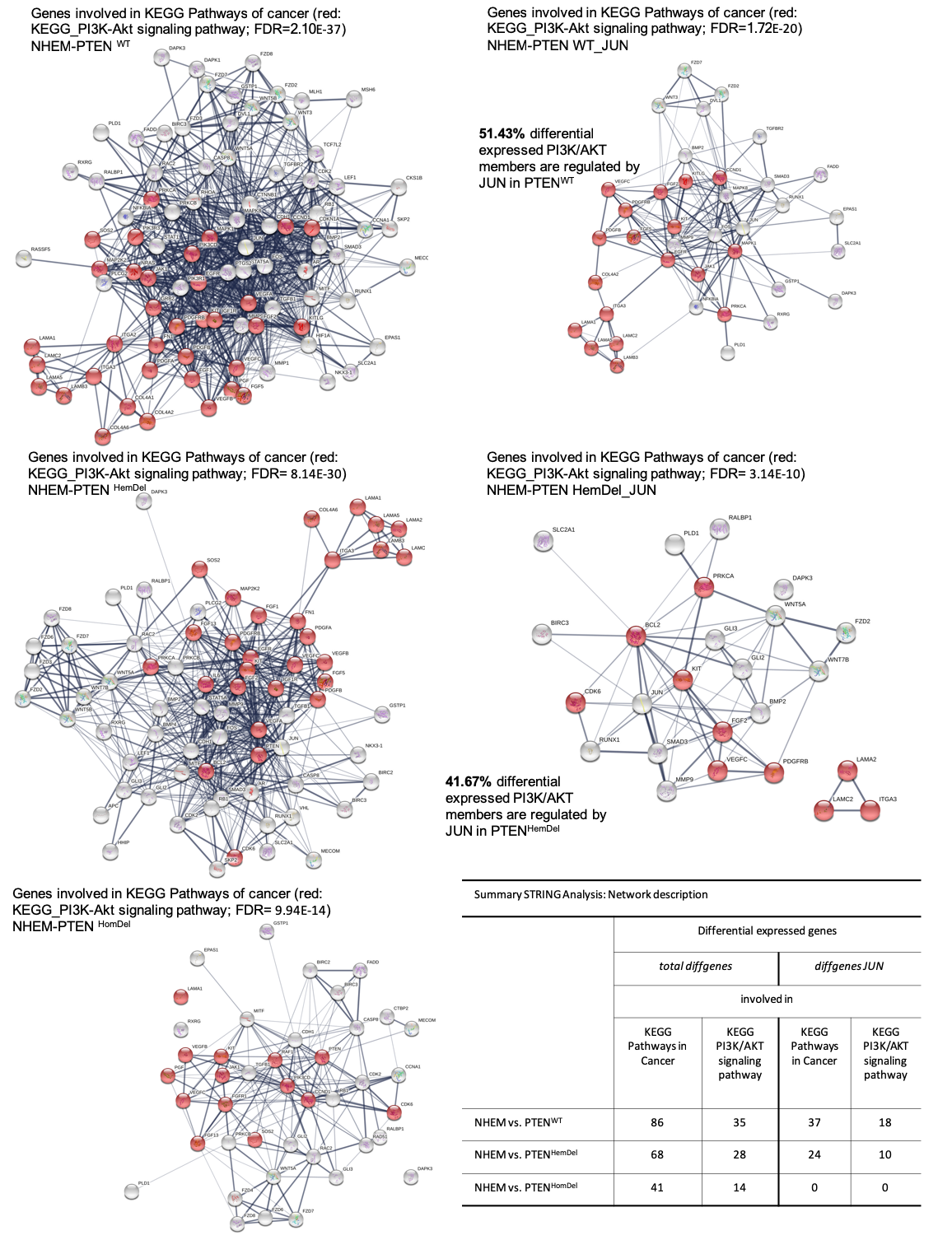


**Supplement Figure 5. String interaction networks of differential expressed genes involved in KEGG *Pathways of cancer*.** Differential expressed genes involved in KEGG Pathways of cancer in *PTEN*^WT^, *PTEN*^HemDel^ and *PTEN*^HomDel^ (left column) and differential expressed genes regulated by c-Jun involved in KEGG Pathways of cancer in *PTEN*^WT^_ c-Jun or *PTEN*^HemDel^_ c-Jun (right column). PI3K/AKT signaling members are depicted in red.

**Supplement Figure 6. siPool transfection efficiencies. (A)** Western blot analysis of sic-Jun-pool transfected WM1366 melanoma cells. ß-Actin served as loading control. **(B)** PTEN mRNA expression analysis via qRT-PCR of siPTEN-pool transfected NHEMs and WM1366 normalized to ß-actin. **(C)** AKT1 and AKT3 mRNA expression analysis via qRT-PCR of siAKT-pool transfected WM1366 and WM9 normalized to ß-actin. The bars show the mean ±s.d. of three independent experiments; (*P<0.05).

**Supplementary References**

1 Todorovic, V. *et al.* Metastatic potential of melanoma cells is not affected by electrochemotherapy. *Melanoma research* **21**, 196-205, (2011).

2 Zaiss, Dietmar M. W. *et al.* Amphiregulin Enhances Regulatory T Cell-Suppressive Function via the Epidermal Growth Factor Receptor. *Immunity* **38**, 275-284, (2013).

3 Wu, X. *et al.* c-Met, epidermal growth factor receptor, and insulin-like growth factor-1 receptor are important for growth in uveal melanoma and independently contribute to migration and metastatic potential. *Melanoma research* **22**, 123-132, (2012).

4 Collisson, E. A. *et al.* Atorvastatin prevents RhoC isoprenylation, invasion, and metastasis in human melanoma cells. *Molecular cancer therapeutics* **2**, 941-948, (2003).

5 Reiland, J., Kempf, D., Roy, M., Denkins, Y. & Marchetti, D. FGF2 Binding, Signaling, Angiogenesis Are Modulated by Heparanase in Metastatic Melanoma Cells. *Neoplasia* **8**, 596-606, (2006).

6 Chalkiadaki, G. *et al.* Fibroblast growth factor-2 modulates melanoma adhesion and migration through a syndecan-4-dependent mechanism. *The international journal of biochemistry & cell biology* **41**, 1323-1331, (2009).

7 Barnhill, R. L., Xiao, M., Graves, D. & Antoniades, H. N. Expression of platelet-derived growth factor (PDGF)-A, PDGF-B and the PDGF-alpha receptor, but not the PDGF-beta receptor, in human malignant melanoma in vivo. *British Journal of Dermatology* **135**, 898-904, (1996).

8 Qiu, T. *et al.* Identification of genes associated with melanoma metastasis. *Kaohsiung J Med Sci* **31**, 553-561, (2015).

9 Lund, Amanda W. *et al.* VEGF-C Promotes Immune Tolerance in B16 Melanomas and Cross-Presentation of Tumor Antigen by Lymph Node Lymphatics. *Cell Reports* **1**, 191-199, (2012).

10 Peppicelli, S., Bianchini, F., Contena, C., Tombaccini, D. & Calorini, L. Acidic pH via NF-κB favours VEGF-C expression in human melanoma cells. *Clinical & experimental metastasis* **30**, 957-967, (2013).

11 Rovenska, E. [Importance of lymphangiogenesis and ultrastructure of lymphatic capillaries in metastasis of malignant melanoma]. *Vnitr Lek* **60**, 582-585, (2014).

12 Smith, S. D. *et al.* Protein kinase Calpha (PKCalpha) regulates p53 localization and melanoma cell survival downstream of integrin alphav in three-dimensional collagen and in vivo. *The Journal of biological chemistry* **287**, 29336-29347, (2012).

13 Halder, K., Banerjee, S., Bose, A., Majumder, S. & Majumdar, S. Overexpressed PKCdelta downregulates the expression of PKCalpha in B16F10 melanoma: induction of apoptosis by PKCdelta via ceramide generation. *PloS one* **9**, e91656, (2014).

14 Krasagakis, K. *et al.* Proliferation of human melanoma cells is under tight control of protein kinase C alpha. *Journal of cellular physiology* **199**, 381-387, (2004).

15 Papandreou, C. *et al.* Mutation and expression of the low affinity neurotrophin receptor in human malignant melanoma. *Melanoma research* **6**, 373-378, (1996).

16 Ghassemi, S. *et al.* FGF5 is expressed in melanoma and enhances malignancy <i>in vitro</i> and <i>in vivo</i>. *Oncotarget* **8**, (2017).

17 Yoshinaga, I. G., Vink, J., Dekker, S. K., Mihm, M. C. & Byers, H. R. Role of α3β1 and α2β1 integrins in melanoma cell migration. *Melanoma research* **3**, 435-442, (1993).

18 Sun, C. *et al.* Reversible and adaptive resistance to BRAF(V600E) inhibition in melanoma. *Nature* **508**, 118-122, (2014).

19 McGary, E. C. *et al.* Imatinib mesylate inhibits platelet-derived growth factor receptor phosphorylation of melanoma cells but does not affect tumorigenicity in vivo. *The Journal of investigative dermatology* **122**, 400-405, (2004).

20 Etoh, T., Byers, H. R. & Mihm, M. C. Integrin Expression in Malignant Melanoma and Their Role in Cell Attachment and Migration on Extracellular Matrix Proteins. *The Journal of Dermatology* **19**, 841-846, (1992).

21 Kim, H. *et al.* Downregulation of the Ubiquitin Ligase RNF125 Underlies Resistance of Melanoma Cells to BRAF Inhibitors via JAK1 Deregulation. *Cell Reports* **11**, 1458-1473, (2015).

22 Mirmohammadsadegh, A. *et al.* STAT5 Phosphorylation in Malignant Melanoma Is Important for Survival and Is Mediated Through SRC and JAK1 Kinases. *Journal of Investigative Dermatology* **126**, 2272-2280, (2006).

23 Luan, Q. *et al.* RIPK1 regulates survival of human melanoma cells upon endoplasmic reticulum stress through autophagy. *Autophagy* **11**, 975-994, (2015).

24 Nagatsuka, H. *et al.* Immunolocalization and distribution patterns of type IV collagen alpha chains in oral mucosal melanoma. *Virchows Archiv* **447**, 710-716, (2005).

25 Vízkeleti, L. *et al.* The role of CCND1 alterations during the progression of cutaneous malignant melanoma. *Tumor Biology* **33**, 2189-2199, (2012).

26 Postow, M. A. & Carvajal, R. D. Therapeutic implications of KIT in melanoma. *Cancer J* **18**, 137-141, (2012).

27 Tran, A. & Tawbi, H. A. A potential role for nilotinib inKIT-mutated melanoma. *Expert opinion on investigational drugs* **21**, 861-869, (2012).

28 Pho, L. N. & Leachman, S. A. Genetics of pigmentation and melanoma predisposition. *G Ital Dermatol Venereol* **145**, 37-45, (2010).

29 Yoshinaga, I. G., Vink, J., Dekker, S. K., Mihm, M. C., Jr. & Byers, H. R. Role of alpha 3 beta 1 and alpha 2 beta 1 integrins in melanoma cell migration. *Melanoma research* **3**, 435-441, (1993).

30 Byers, H. R., Boissel, S. J., Tu, C. & Park, H. Y. RNAi-mediated knockdown of protein kinase C-alpha inhibits cell migration in MM-RU human metastatic melanoma cell line. *Melanoma research* **20**, 171-178, (2010).

31 Eberle, J., Krasagakis, K., Garbe, C. & Orfanos, C. E. Proliferation and morphology of melanoma cells and benign human melanocytes under varying culture conditions. *Melanoma research* **3**, 107-112, (1993).

32 Goydos, J. S. & Gorski, D. H. Vascular endothelial growth factor C mRNA expression correlates with stage of progression in patients with melanoma. *Clinical cancer research : an official journal of the American Association for Cancer Research* **9**, 5962-5967, (2003).

33 Boone, B. *et al.* The role of VEGF-C staining in predicting regional metastasis in melanoma. *Virchows Archiv : an international journal of pathology* **453**, 257-265, (2008).

34 Spofford, L. S., Abel, E. V., Boisvert-Adamo, K. & Aplin, A. E. Cyclin D3 expression in melanoma cells is regulated by adhesion-dependent phosphatidylinositol 3-kinase signaling and contributes to G1-S progression. *The Journal of biological chemistry* **281**, 25644-25651, (2006).

35 Miglarese, M. R., Halaban, R. & Gibson, N. W. Regulation of fibroblast growth factor 2 expression in melanoma cells by the c-MYB proto-oncoprotein. *Cell growth & differentiation : the molecular biology journal of the American Association for Cancer Research* **8**, 1199-1210, (1997).

36 Ribatti, D. *et al.* Neovascularisation, expression of fibroblast growth factor-2, and mast cells with tryptase activity increase simultaneously with pathological progression in human malignant melanoma. *European journal of cancer* **39**, 666-674, (2003).

37 Kramer, R. H. *et al.* Laminin-binding integrin alpha 7 beta 1: functional characterization and expression in normal and malignant melanocytes. *Cell Regul* **2**, 805-817, (1991).

38 Natali, P. G., Nicotra, M. R., Cavaliere, R., Giannarelli, D. & Bigotti, A. Tumor progression in human malignant melanoma is associated with changes in alpha 6/beta 1 laminin receptor. *International journal of cancer. Journal international du cancer* **49**, 168-172, (1991).

39 Hartman, M. L. & Czyz, M. Anti-apoptotic proteins on guard of melanoma cell survival. *Cancer letters* **331**, 24-34, (2013).
